# Novel series of 1,2,4-trioxane derivatives as antimalarial agents

**DOI:** 10.1080/14756366.2017.1363742

**Published:** 2017-09-05

**Authors:** Mithun Rudrapal, Dipak Chetia, Vineeta Singh

**Affiliations:** a Department of Pharmaceutical Sciences, Dibrugarh University, Dibrugarh, India;; b Parasite Bank, National Institute of Malaria Research (ICMR), Sector 8, Dwarka, New Delhi, India

**Keywords:** *P. falciparum*, resistance, trioxane derivatives, falcipain 2 inhibitors, antimalarial

## Abstract

Among three series of 1,2,4-trioxane derivatives, five compounds showed good *in vitro* antimalarial activity, three compounds of which exhibited better activity against *P. falciparum* resistant (RKL9) strain than the sensitive (3D7) one. Two best compounds were one from aryl series and the other from heteroaryl series with IC_50_ values of 1.24 µM and 1.24 µM and 1.06 µM and 1.17 µM, against sensitive and resistant strains, respectively. Further, trioxane derivatives exhibited good binding affinity for the *P. falciparum* cysteine protease falcipain 2 receptor (PDB id: 3BPF) with well defined drug-like and pharmacokinetic properties based on Lipinski’s rule of five with additional physicochemical and ADMET parameters. In view of having antimalarial potential, 1,2,4-trioxane derivative(s) reported herein may be useful as novel antimalarial lead(s) in the discovery and development of future antimalarial drug candidates as *P. falciparum* falcipain 2 inhibitors against resistant malaria.

## Introduction

Malaria continues to be a major lethal infectious disease of human beings, affecting around 200–300 million people and causing approximately 430,000 deaths every year globally. The most affected populations are children and infants as well as pregnant women^1^. The disease is caused by five species of the parasite *Plasmodium*, namely, *Plasmodium falciparum*, *Plasmodium vivax*, *Plasmodium ovale*, *Plasmodium malariae*, and *Plasmodium knowlesi*. Of these, *P. falciparum* is the most prevalent and deadly species that causes severe malaria such as cerebral malaria, and it is also responsible for most of the malaria-related deaths in humans^2–4^. Every year over 50% of all malarial infections are caused by *P. falciparum* with about 20–50% of total mortality cases commonly occur in children younger than five years old and pregnant women^5^. During the past two decades, the emergence of drug resistant strains of *P. falciparum* has become an increasingly serious concern in malaria control and prevention worldwide. Reduced clinical efficacy of currently available drugs, especially those in the aminoquinoline group (chloroquine, amodiaquine, mefloquine, and piperaquine) including non-quinolines like antifolates (sulfadoxine, proguanil, pyrimethamine), lumefantrine, atovaquone, and antibiotics, and the novel artemisinin (ART) derivatives (dihydroartemisinin, artemether, and artesunate), to multi-drug resistant *P. falciparum* strains has limited their clinical usefulness in the treatment of resistant malaria^2^. Artemisinin-based combination therapies (ACTs) such as artemether/lumefantrine/amodiaquine and artesunate/mefloquine/piperaquine recommended by WHO for the treatment of multi-drug resistant *P. falciparum* malaria have also been reported to develop resistance in South-East Asia^2^ ^,^ ^6^. This increasing burden of resistant malaria has stimulated drug discovery scientists to search for new antimalarial drugs or alternative therapeutic options to combat the problem of drug resistance. In view of this, the development of new antimalarial drugs with novel mode(s) of action could be an attractive strategy to address the above challenging issue.

**Figure 1.**
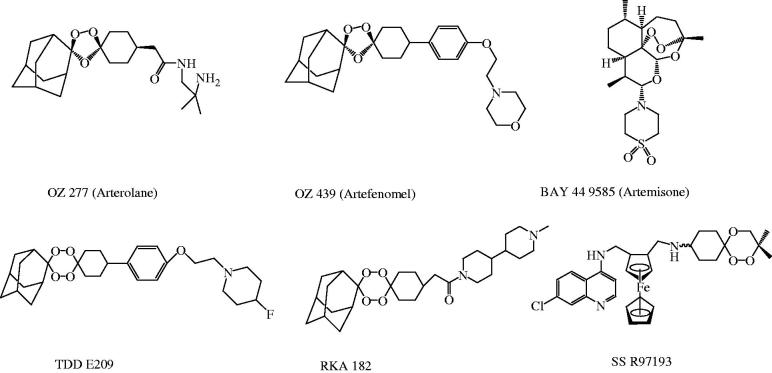
Structures of some new endoperoxide antimalarials.

Though ART and related endoperoxides are the mainstay of current antimalarial therapy, high treatment cost (relative to quinoline-based drugs), unsatisfactory physicochemical/pharmacokinetic properties, toxicities and limited availability of drugs are some other notable pitfalls besides having potential resistance problem^7^ ^,^ ^8^. All above issues are adversely affecting clinical outcomes of ART-based drug regimens used in the treatment of malaria. The clinical significance of ART-based drugs is attributed to be due to the excellent antiparasitic action of the endoperoxide component. Pharmacodynamic study also suggests that the key pharmacophoric 1,2,4-trioxane ring system is responsible for the biological activity of ART (natural lead molecule), its semi-synthetic analogues and related endoperoxides^6^ ^,^ ^9^ ^,^ ^10^. In recent years, research into several synthetic endoperoxide scaffolds such as 1,2,4-trioxane, 1,2,4-trioxolane and 1,2,4,5-teraoxane derivatives and their hybrid analogues have gained significant interest in discovery and development of potent antimalarial drugs. Some endoperoxide-based antimalarial compounds^2^ that are currently under clinical phase of development are represented in Figure 1. Endoperoxide molecules bearing 1,2,4-trioxane core have been identified to exhibit a broad spectrum of biological effects such as anticancer, antimicrobial, and anti-parasitic effects^11^. Several design strategies have been initiated by MMV (Medicines for Malaria Venture) program for the development of effective and affordable synthetic peroxides as alternative therapy to existing ART-based drugs^2^ ^,^ ^12^. However, considering all above facts, development of peroxide-based synthetic compounds is expected to provide effective antimalarials agents with limited toxicity issues and ready to be available at affordable cost. It would offer a better therapeutic option for the treatment of malaria with good clinical outcomes (desired potency with optimal pharmacokinetics) and resistant preventing action. It may therefore represent a new class of synthetic, new-generation and orally active peroxides, with optimal antimalarial potency against resistant *P. falciparum* malaria.

As a part of our research program towards developing potent antimalarial drugs that would be active against resistant *P. falciparum* malaria, novel molecular manipulation approach was adopted to design and develop newer series of 1,2,4-trioxane derivatives as single drug conjugates (single molecular entity rather than hybrid drugs) based on the basic structural scaffold of 1,2,4-trioxane ring system. After the synthesis of compounds, *in vitro* antimalarial screening, molecular docking and drug-likeness studies including ADMET prediction using *in silico* tools were also carried out. Since, ART and its semi-synthetic analogues or ART derived peroxides possess unfavourable pharmacokinetic issues and certain tissue toxicities because of their high structural complexities and some physicochemical insufficiencies^7^ ^,^ ^8^, it is expected that designed compounds would possess modified pharmacokinetic properties and toxicities due to less complexities in the molecular structure. Moreover, single drug conjugate-based trioxane molecule can be given as single drug therapy that would also be advantageous over hybrid antimalarial drugs and combination therapies.

Molecular docking study was performed to validate the antimalarial efficacy of synthesised 1,2,4-trioxane derivatives by investigating binding modes as well as orientation of ligands in the receptor pocket of target falcipain 2 protein (*P. falciparum* cysteine protease enzyme). In our study, three-dimensional (3D) structure of falcipain 2 protein molecule was used as possible antimalarial drug target for 1,2,4-trioxane derivatives. Docking rationalises finding new antimalarial lead molecules and also gives an insight into structure–activity relationships (SARs) and mode(s) of antimalarial action based on scoring function and further binding modes analysis from ligand–protein binding/interaction studies^13^. Drug-likeness study includes calculation of fundamental molecular (physicochemical) properties and Lipinski’s parameters, which assesses the acceptability of compounds as drug molecules. They represent the combined physicochemical, pharmacokinetic and pharmacodynamic properties (collectively termed as drug-like properties) of molecules to exhibit good drug-likeness behaviour in human body^14^. In fact, molecular properties are the fundamental structural parameters which determine the physicochemical (solubility and permeability) and biochemical (metabolic stability, transport property, and protein/tissue affinity) properties, which ultimately determine molecule’s *in vivo* pharmacokinetics (bioavailability and half life), toxicity, and pharmacodynamics (receptor affinity and efficacy)^15^. ADMET (absorption, distribution, metabolism, excretion, and toxicity) properties have a predictable influence on pharmacokinetic and pharmacodynamic effects of drug molecules. The calculation of ADMET properties is therefore intended as the first step towards optimising the new drug molecules, whether to check the failure of lead molecules which may cause toxicity and one unable to cross the intestinal membranes or metabolised by the body in to an inactive form^16^.

## Experimental

### Synthesis and analysis

All of the chemicals were procured commercially from Sigma-Aldrich Corporation (St. Louis, MO), Merck Specialists Pvt. Ltd. (Darmstadt, Germany), HiMedia Lab. Pvt. Ltd. (Einhausen, Germany), or Spectrochem Pvt. Ltd. (Mumbai, India) and were used without further purification. All the reactions were performed in oven dried glass ware using synthetic grade chemicals. Melting points (MP) were measured in open capillaries on an electrically heated MP apparatus. Ultraviolet (UV)–visible spectra were recorded on Shimadzu UV-1700 UV–visible spectrophotometer and the wave lengths of maximum absorption (*λ*_max_, nm) are reported. Infrared (IR) spectra were obtained on a Bruker Alpha Fourier Transform (FT-IR) spectrophotometer (Billerica, MA) using KBR disc and are reported in terms of frequency of absorption (*υ*, cm^−1^). ^1^H and ^13^C nuclear magnetic resonance (NMR) spectra were recorded on Bruker Avance II 400 FT-NMR spectrometer (Billerica, MA) at 400 and 100 MHz, respectively, using tetramethylsilane (TMS) as an internal standard (*δ* 0.00 ppm) and CDCl_3_ as a solvent. Chemical shift (*δ*) values were expressed in parts per million (ppm) relative to TMS (*δ* 0.00 ppm). ^1^HNMR data are assigned in order: peak multiplicity (s, singlet; d, doublet; t, triplet; m, multiplet), number of protons (numerical integral value), coupling constants (*J* value) in hertz. ^13^C NMR data represents different types of structural carbons with respect to their corresponding chemical shift values. Mass spectra were obtained on a LC–MS Water 4000 ZQ instrument using electrospray ionisation (ES^+^). The *m/z* values were recorded in the range of *m/z* between 100 and 500 and the *m/z* values of the most intense molecular ion [M]^+^ peak, with relative intensities in parentheses, are given followed by peaks corresponding to major fragment ions. Elemental analysis was performed on a ThermoFinnigan CHNS Analyzer (Somerset, NJ).

## General procedure of synthesis^11^ ^,^ ^17^, 3a–e, 3′a–n, 3″a–e

A mixture of *p*-cresol, **1** (1 mmol, 0.1045 ml), distilled water (16.0 ml) and acetonitrile (CH_3_CN, 4.0 ml) was vigorously stirred until complete solution was achieved. A mixture of oxone (5 mmol, 1.537 g) and NaHCO_3_ (15 mmol, 1.261 g), previously ground into powder, was slowly added in portion, and a septum with an empty balloon was immediately placed into the flask in order to avoid overpressure and loss of generated singlet oxygen. The mixture was vigorously stirred at room temperature until total disappearance of the phenol. After about 1 h of stirring, the reaction was quenched with water and extracted several times (3–4 times) with ethyl acetate (EtOAc). Combined EtOAc extracts were dried in Na_2_SO_4_ and the solvent was removed under reduced pressure to yield the product of *p*-peroxyquinol, **2**. The crude compound was recrystallised from EtOAc/ethanol mixture (3:1) to obtain a pure product of white crystalline solid.

The intermediate compound, *p*-peroxyquinol (4-methyl-4-hydroperoxycyclohexa-2,5-dienone) **2** (1 mmol) was added slowly into a mixture of aldehyde (1.5 mmol) and dichloromethane (4.0 ml), followed by the addition of an acid catalyst, *p*-toluenesulfonic acid (PTSA, 0.05 mmol). The reaction mixture was stirred at 45–5 °C till the completion of reaction (8–12 h) and then concentrated in vacuum. Colum chromatography (silica gel 60, 230–400 mesh) of the resulting residue was carried out using a solvent system of hexane:EtOAc (80:20) to obtain the desired pure product of target compounds, **3a–e**, **3′a–n**, **3″a–e** as crystalline solids or semi-solids.

### 3-Ethyl-8a-methyl-4a,5-dihydrobenzo-1,2,4-trioxin-6(8aH)-one (3a)

UV spectrum (MeOH), *λ*_max_, nm: 212.8; IR (KBr, cm^−1^) *υ*: 2940.31, 2883.27 (C–H, CH_3_), 1656.48 (C=O, conj.), 1145.20 (C–O), 892.28 (O–O); ^1^H NMR (400 MHz, CDCl_3_) *δ*: 0.89 (t, *J* = 6.0 Hz, 3H, CH_3_), 1.21 (s, 3H, CH_3_), 1.43 (q, *J* = 6.0 Hz, 3H, CH_2_–CH_3_), 2.46 (d, *J* = 3.0 Hz, 2H, CO–CH_2_–CH), 4.12 (d, *J* = 3.0 Hz, 1H, CO–CH_2_–CH), 5.24 (t, *J* = 6.0 Hz, 1H, CH–CH_2_–CH_3_), 6.04 (d, *J* = 9.0, 1H, CO–CH=CH), 6.32 (d, *J* = 9.0, 1H, CO–CH=CH); ^13^C NMR (100 MHz, CDCl_3_) *δ*: 14.10, 18.24 (CH_3_), 22.90, 26.23 (CH_2_), 36.81, 42.37 (CH), 84.77, 92.40 (CH=CH), 188.27 (C=O); MS (ES^+^), *m/z* (%): 198.78 (100), [M]^+^, 182.12 (52), 176.12 (28), 152.38 (6); CHN anal. for C_10_H_14_O_4_ (198.22), calc. (%): C, 60.59, H, 7.12, O, 32.29, found (%): C, 61.09, H, 6.72, O, 31.69.

### 3-Butyl-8a-methyl-4a,5-dihydrobenzo-1,2,4-trioxin-6(8aH)-one (3c)

UV spectrum (MeOH), *λ*_max_, nm: 218.6; IR (KBr, cm^−1^) *υ*: 2923.67, 2888.36 (C–H, CH_3_), 1668.59 (C=O, conj.), 1177.34 (C–O), 886.56 (O–O); ^1^H NMR (400 MHz, CDCl_3_) *δ*: 0.92 (t, *J* = 6.0 Hz, 3H, CH_3_), 1.18 (s, 3H, CH_3_), 1.46 (m, 2H, CH_2_–CH_2_–CH_3_), 1.52 (m, 2H, CH_2_–CH_2_–CH_3_), 1.63 (m, 2H, CH_2_–CH_2_–CH_2_–CH_3_), 2.52 (d, *J* = 3.0 Hz, 2H, CO–CH_2_–CH), 4.23 (d, *J* = 3.0 Hz, 1H, CO–CH_2_–CH), 6.06 (t, *J* = 6.0 Hz, 1H, CH–CH_2_–CH_3_), 6.09 (d, *J* = 10.0, 1H, CO–CH=CH), 6.34 (d, *J* = 9.0, 1H, CO–CH=CH); ^13^C NMR (100 MHz, CDCl_3_) *δ*: 15.53, 18.18 (CH_3_), 22.43, 28.10, 32.62 (CH_2_), 36.36, 46.21 (CH), 86.54, 93.26 (CH=CH), 187.60 (C=O); MS (ES^+^), *m/z* (%): 226.27 (100), [M]^+^, 182.12 (52), 176.12 (28), 152.38 (6); CHN anal. for C_12_H_18_O_4_ (198.22), calc. (%): C, 63.70, H, 8.02, O, 28.28, found (%): C, 64.42, H, 8.71, O, 27.78.

### 3-(2-Hydroxyphenyl)-8a-methyl-4a,5-dihydrobenzo-1,2,4-trioxin-6(8aH)-one (3′b)

UV spectrum (MeOH), *λ*_max_, nm: 284.2; IR (KBr, cm^−1^) *υ*: 3174.10 (O–H, bonded), 2970.21, 2879.23 (C–H, CH_3_), 1667.13 (C=O, conj.), 1595.12, 1514.23, 1423.14 (C=C, aryl), 1161.15 (C–O), 832.19 (O–O); ^1^H NMR (400 MHz, CDCl_3_) *δ*: 1.25 (s, 3H, CH_3_), 7.01 (1H, CO–CH=CH), 7.02 (1H, CO–CH=CH), 7.82 (Ar–H), 7.84 (Ar–H), 9.83 (Ar–OH); ^13^C NMR (100 MHz, CDCl_3_) *δ*: 15.42 (CH_3_), 29.72 (CH), 116.02, 129.69, 132.55, 161.79 (Ar–C), 191.34 (C=O); MS (ES^+^), *m/z* (%): 262.26 (100), [M]^+^, 230.38 (59), 192.37 (34), 176.03 (21); CHN anal. for C_14_H_14_O_5_ (262.26), calc. (%): C, 64.12, H, 5.38, O, 30.50, found (%): C, 64.62, H, 4.78, O, 30.00.

### 3-(3-Methoxyphenyl)-8a-methyl-4a,5-dihydrobenzo-1,2,4-trioxin-6(8aH)-one (3′c)

UV spectrum (MeOH), *λ*_max_, nm: 292.3; IR (KBr, cm^−1^) *υ*: 2956.33, 2880.16 (C–H, CH_3_), 1674.20 (C=O, conjugated), 1642.13, 1582.30, 1424.10 (C=C, aryl), 1152.82 (C–O), 878.41 (O–O); ^1^H NMR (400 MHz, CDCl_3_) *δ*: 1.19 (s, 3H, CH_3_), 2.43 (d, *J* = 4.0 Hz, 2H, CO–CH_2_–CH), 4.02 (s, 3H, OCH_3_), 4.23 (d, *J* = 4.0 Hz, 1H, CO–CH_2_–CH), 6.04 (d, *J* = 10.0, 1H, CO–CH=CH), 6.37 (d, *J* = 10.0, 1H, CO–CH=CH), 7.03 (d, 1H, *J* = 7.0 Hz, Ar–H), 7.28 (d, *J* = 7.0 Hz, Ar–H), 7.44 (d, *J* = 8.0 Hz, Ar–H), 7.82 (d, 1H, *J* = 8.0 Hz, Ar–H); ^13^C NMR (100 MHz, CDCl_3_) *δ*: 15.42 (CH_3_), 29.12 (OCH_3_), 38.40 (CH), 84.65, 92.28 (CH=CH), 118.18, 126.12, 132.17, 140.72, 152.32, 162.25 (Ar–C), 190.20 (C=O); MS (ES^+^), *m/z* (%): 276.48 (100), [M]^+^, 223.01 (64), 182.98 (32), 142.04 (18); CHN anal. for C_15_H_16_O_5_ (276.28), calc. (%): C, 65.21, H, 5.84, O, 28.95, found (%): C, 65.72, H, 6.24, O, 28.55.

### 3-(4-Chlorophenyl)-8a-methyl-4a,5-dihydrobenzo-1,2,4-trioxin-6(8aH)-one (3′d)

UV spectrum (MeOH), *λ*_max_, nm: 255.0; IR (KBr, cm^−1^) *υ*: 2912.56, 2898.20 (C–H, CH_3_), 1672.17 (C=O, conj.), 1142.10 (C–O), 1054.36 (Ar. C–Cl), 878.12 (O–O); ^1^H NMR (400 MHz, CDCl_3_) *δ*: 1.16 (s, 3H, CH_3_), 2.41 (d, *J* = 4.0 Hz, 2H, CO–CH_2_–CH), 4.22 (d, *J* = 4.0 Hz, 1H, CO–CH_2_–CH), 6.06 (d, *J* = 10.0, 1H, CO–CH=CH), 6.34 (d, *J* = 10.0, 1H, CO–CH=CH), 7.12 (d, 1H, *J* = 8.0 Hz, Ar–H), 7.46 (d, *J* = 8.0 Hz, Ar–H); ^13^C NMR (100 MHz, CDCl_3_) *δ*: 15.47 (CH_3_), 36.38 (CH), 89.42, 94.17 (CH=CH), 117.20, 128.13, 134.60, 140.44, 152.02, 160.12 (Ar–C), 188.00 (C=O); MS (ES^+^), *m/z* (%): 262.71 (100), [M]^+^, 242.12 (62), 222.13 (18), 154.72 (6); CHN anal. for C_14_H_13_O_4_Cl (280.70), calc. (%): C, 59.90, H, 4.67, O, 22.80, found (%): C, 60.50, H, 4.07, O, 20.41.

### 3-(4-Nitrorophenyl)-8a-methyl-4a,5-dihydrobenzo-1,2,4-trioxin-6(8aH)-one (3′e)

UV spectrum (MeOH), *λ*_max_, nm: 265.0; IR (KBr, cm^−1^) *υ*: 2932.21, 2887.12 (C–H, CH_3_), 1664.20 (C=O, conj.), 1543.69 (N–O, NO_2_), 1346 (N–O, NO_2_), 1142.78 (C–O), 873.11 (O–O); ^1^H NMR (400 MHz, CDCl_3_) *δ*: 1.20 (s, 3H, CH_3_), 2.44 (d, *J* = 3.0 Hz, 2H, CO–CH_2_–CH), 4.62 (d, *J* = 3.0 Hz, 1H, CO–CH_2_–CH), 6.13 (d, *J* = 10.0, 1H, CO–CH=CH), 6.38 (d, *J* = 10.0, 1H, CO–CH=CH), 7.24 (d, 1H, *J* = 8.0 Hz, Ar–H), 7.74 (d, *J* = 8.0 Hz, Ar–H); ^13^C NMR (100 MHz, CDCl_3_) *δ*: 14.13 (CH_3_), 39.11 (CH), 86.70, 90.12 (CH=CH), 116.12, 124.34, 132.26, 138.25, 147.10, 158.33 (Ar–C), 192.12 (C=O); MS (ES^+^), *m/z* (%): 291.45 (100), [M]^+^, 272.11 (52), 237.18 (27), 192.16 (12); CHN anal. for C_14_H_13_NO_6_ (291.26), calc. (%): C, 57.73, H, 4.50, N, 4.81, O, 32.96, found (%): C, 58.32, H, 4.10, N, 4.21, O, 32.16.

### 3-(4-(Dimethylamino)phenyl)-8a-methyl-4a,5-dihydrobenzo-1,2,4-trioxin-6(8aH)-one (3′j)

UV spectrum (MeOH), *λ*_max_, nm: 242.0, 344.0; IR (KBr, cm^−1^) *υ*: 2928.15, 2876.42 (C–H, CH_3_), 1664.74 (C=O, conj.), 1623.16, 1590.12, 1482.49 (C=C, aryl), 1278.32 (C–N), 1164.23 (C–O), 846.45 (O–O); ^1^H NMR (400 MHz, CDCl_3_) *δ*: 1.22 (s, 3H, CH_3_), 2.18 (d, *J* = 3.0 Hz, 2H, CO–CH_2_–CH), 2.82 (d, *J* = 12.0 Hz, 3H, N–(CH_3_)_2_), 2.96 (d, *J* = 12.0 Hz, 3H, N–(CH_3_)_2_), 4.24 (d, *J* = 3.0 Hz, 1H, CO–CH_2_–CH), 4.88 (t, *J* = 7.0 Hz, 1H, CH–CH_2_–CH_3_), 6.23 (d, *J* = 9.0, 1H, CO–CH=CH), 6.36 (d, *J* = 9.0, 1H, CO–CH=CH), 7.22 (d, 1H, *J* = 8.0 Hz, Ar–H), 7.68 (d, *J* = 8.0 Hz, Ar–H); ^13^C NMR (100 MHz, CDCl_3_) *δ*: 14.25, 15.60, 18.13 (CH_3_), 38.03 (CH), 86.40, 89.34 (CH=CH), 116.45, 128.02, 132.57, 136.44, 150.13, 160.11 (Ar–C), 190.34 (C=O); MS (ES^+^), *m/z* (%): 289.56 (100), [M]^+^, 262.22 (60), 232.17 (28), 182.03 (11); CHN anal. for C_16_H_19_NO_4_ (289.33), calc. (%): C, 66.42, H, 6.62, N, 4.84, O, 22.12, found (%): C, 66.82, H, 6.12, N, 4.98, O, 21.52.

### 3-(4-Hydroxy-3-methoxyphenyl)-8a-methyl-4a,5-dihydrobenzo-1,2,4-trioxin-6(8aH)-one (3′l)

UV spectrum (MeOH), *λ*_max_, nm: 277.6, 305.4; IR (KBr, cm^−1^) *υ*: 3269.19 (O–H, bonded), 3089.21 (C–H, aryl), 2972.24, 2944.25 (C–H, CH_3_), 1679.11 (C=O, conj.), 1642.14, 1593.10, 1510.14 (C=C, aryl), 1043.31 (C–O), 820.17 (O–O); ^1^H NMR (400 MHz, CDCl_3_) *δ*: 1.18 (s, 3H, CH_3_), 3.94 (s, 3H, OCH_3_), 7.03 (1H, CO–CH=CH), 7.05 (1H, CO–CH=CH), 7.42 (Ar–H), 7.43 (Ar–H), 9.81 (Ar–OH); ^13^C NMR (100 MHz, CDCl_3_) *δ*: 56.08 (OCH_3_), 108.84, 114.47, 127.61, 129.73, 147.23, 151.84 (Ar–C), 191.11 (C=O); MS (ES^+^), *m/z* (%): 292.80 (100), [M]^+^, 266.34 (62), 218.15 (36), 178.36 (12); CHN anal. for C_15_H_16_O_6_ (292.28), calc. (%): C, 61.64, H, 5.52, O, 32.84, found (%): C, 62.04, H, 5.93, O, 32.26.

### 3-(3-Formylphenyl)-8a-methyl-4a,5-dihydrobenzo-1,2,4-trioxin-6(8aH)-one (3′n)

UV spectrum (MeOH), *λ*_max_, nm: 226.4, 244.6; IR (KBr, cm^−1^) *υ*: 2932.12, 2882.00 (C–H, CH_3_), 2812.65, 2773.26 (C–H, CHO), 1672.45 (C=O, conjugated), 1632.12, 1568.23, 1534.00, 1482.34 (C=C, aryl), 1163.67 (C–O), 879.34 (O–O); ^1^H NMR (400 MHz, CDCl_3_) *δ*: 1.34 (s, 3H, CH_3_), 2.19 (d, *J* = 3.0 Hz, 2H, CO–CH_2_–CH), 4.24 (d, *J* = 3.0 Hz, 1H, CO–CH_2_–CH), 6.13 (d, *J* = 10.0, 1H, CO–CH=CH), 6.32 (d, *J* = 10.0, 1H, CO–CH=CH), 7.10 (t, *J* = 8.0 Hz, 1H, Ar–H), 7.23 (d, *J* = 7.0 Hz, 1H, Ar–H), 7.44 (d, *J* = 8.0 Hz, 1H, Ar–H), 7.53 (s, 1H, Ar–H), 9.71 (CHO); ^13^C NMR (100 MHz, CDCl_3_) *δ*: 14.87 (CH_3_), 84.60, 92.30 (CH=CH), 112.43, 116.29, 122.70, 132.67, 148.70, 156.13 (Ar–C), 189.76 (C=O); MS (ES^+^), *m/z* (%): 274.27 (100), [M]^+^, 243.37 (54), 210.00 (32), 189.56 (14); CHN anal. for C_15_H_14_O_5_ (274.27), calc. (%): C, 65.69, H, 5.15, O, 29.17, found (%): C, 66.49, H, 5.34, O, 28.56.

### 3-(1H-pyrrol-2-yl)-8a-methyl-4a,5-dihydrobenzo-1,2,4-trioxin-6(8aH)-one (3″c)

UV spectrum (MeOH), *λ*_max_, nm: 242.6, 259.2; IR (KBr, cm^−1^) *υ*: 3247.01 (NH, 1*H*-pyrrol-3-yl), 2920.12, 2889.12 (C–H, CH_3_), 1657.26 (C=O, conj.), 1123.05 (C–O), 874.11 (O–O); ^1^H NMR (400 MHz, CDCl_3_) *δ*: 1.32 (s, 3H, CH_3_), 2.17 (d, *J* = 3.0 Hz, 2H, CO–CH_2_–CH), 4.26 (d, *J* = 3.0 Hz, 1H, CO–CH_2_–CH), 6.17 (d, *J* = 10.0, 1H, CO–CH=CH), 6.38 (d, *J* = 10.0, 1H, CO–CH=CH), 7.01 (d, *J* = 8.0 Hz, 1H, 1*H*-pyrrole-H_3_), 7.12 (t, 1H, *J* = 8.0 Hz, 1*H*-pyrrole-H_4_), 7.42 (t, 1H, *J* = 8.0 Hz, pyrrole-H_5_), 7.67 (s, 1H, NH, 1*H*-pyrrole-H_1_); ^13^C NMR (100 MHz, CDCl_3_) *δ*: 15.32 (CH_3_), 86.52, 88.46 (CH=CH), 107.23, 114.26, 118.40, 122.35 (Ar–C, 1*H*-pyrrole-2-yl), 186.38 (C=O); MS (ES^+^), *m/z* (%): 235.44 (100), [M]^+^, 182.48 (39), 136.59 (24), 84.72 (7); CHN anal. for C_12_H_13_NO_4_ (235.24), calc. (%): C, 61.27; H, 5.57; N, 5.95; O, 27.21, found (%): C, 62.06, H, 5.22, N, 5.22, O, 26.68.

### 3-(1H-indol-3-yl)-8a-methyl-4a,5-dihydrobenzo-1,2,4-trioxin-6(8aH)-one (3″d)

UV spectrum (MeOH), *λ*_max_, nm: 288.4, 295.8; IR (KBr, cm^−1^) *υ*: 3358.34 (NH, *1H*-indol-3-yl), 3066.28, 3040.28 (C–H, aryl), 2872.17 (C–H, CH_3_), 1694.10 (C=O, conj.), 1481.36, 1450.25 (C=C, conj., aryl), 1144.17 (C–O), 865.45 (O–O); ^1^H NMR (400 MHz, CDCl_3_) *δ*: 1.24 (s, 3H, CH_3_), 7.23 (1H, CO–CH=CH), 7.21 (1H, CO–CH=CH), 7.46 (d, *J* = 7.0 Hz, 1H, *1H*-indole-H_4_), 7.48 (1H, *J* = 7.0 Hz, *1H*-indole-H_5_), 8.08 (1H, *J* = 8.0 Hz, *1H*-indole-H_6_), 8.15 (s, *J* = 9.0 Hz, 1H, *1H*-indole-H_7_), 8.17 (s, 1H, *1H*-indole-H_2_), 9.87 (s, 1H, NH, *1H*-indole-H_1_); ^13^C NMR (100 MHz, CDCl_3_) *δ*: 113.15, 120.08, 122.39, 123.63, 125.02, 125.68, 139.77, (Ar–C, *1H*-indole-3-yl), 187.44 (C=O); MS (ES^+^), *m/z* (%): 285.47 (100), [M]^+^, 253.12 (43), 226.10 (25), 187.19 (14); CHN anal. for C_16_H_15_NO_4_ (285.29), calc. (%): C, 67.36, H, 5.30, N, 4.91, O, 22.43, found (%): C, 67.96, H, 4.70, N, 5.41, O, 17.95.

### 3-(Pyridin-4-yl)-8a-methyl-4a,5-dihydrobenzo-1,2,4-trioxin-6(8aH)-one (3″e)

UV spectrum (MeOH), *λ*_max_, nm: 296.8; IR (KBr, cm^−1^) *υ*: 3023.22, 3010.23 (C–H, aryl), 2932, 2822.11 (C–H, CH_3_), 1667.34 (C=O, conj.), 1481.36, 1450.25 (C=C, conj., aryl), 1154.20 (C–O), 872.13 (O–O); ^1^H NMR (400 MHz, CDCl_3_) *δ*: 1.26 (s, 3H, CH_3_), 2.63 (d, *J* = 4.0 Hz, 2H, CO–CH_2_–CH), 4.32 (d, *J* = 4.0 Hz, 1H, CO–CH_2_–CH), 6.21 (d, *J* = 8.0, 1H, CO–CH=CH), 6.40 (d, *J* = 8.0, 1H, CO–CH=CH), 7.48 (d, *J* = 8.0 Hz, 1H, 4-pyridyl-H_2/6_), 7.68 (1H, *J* = 8.0 Hz, 4-pyridyl-H_3/5_); ^13^C NMR (100 MHz, CDCl_3_) *δ*: 15.65 (CH_3_), 84.72, 92.68 (CH=CH), 116.20, 122.58, 128.34, 140.67 (Ar. C, 4-pyridyl), 188.42 (C=O); MS (ES^+^), *m/z* (%): 247.47 (100), [M]^+^, 204.78 (67), 172.09 (38), 156.20 (24); CHN anal. for C_13_H_13_NO_4_ (247.25), calc. (%): C, 63.15; H, 5.30; N, 5.67; O, 25.88, found (%):C, 63.87; H, 5.06; N, 6.12; O, 25.06.

## Evaluation of antimalarial activity

### Chemicals and parasite strains

All the synthesised compounds, **3a–e**, **3′a–n**, **3″a–e** were evaluated for *in vitro* antimalarial activity at multiple doses against CQ-sensitive (3D7) and CQ-resistant (RKL9) strains of *P. falciparum* using blood (erythrocytic) stage of parasite. The solvents and reagents used in the antimalarial study were of analytical grade and were procured from Sigma-Aldrich Corporation (St. Louis, MO) and HiMedia Lab. Pvt. Ltd. (Einhausen, Germany).

### Assay procedure

The *in vitro* antimalarial activity screening was carried out by microculture Giemsa Stained slide method based on light microscopy. Schizont Maturation Inhibition assay technique was used for the quantitative assessment of parasitaemia and evaluation of drug sensitivity. The assay was conducted using the blood parasite of *in vitro P. falciparum* culture according to the method described by Trager and Jensen^18^ ^,^ ^19^. Briefly, a continuous culture of *P. falciparum* strain was maintained *in vitro* in A^+^ human red blood cells (RBCs) diluted to 5% haematocrit (HCT) in RPMI (Roswell Park Memorial Institute)-1640 medium supplemented with 25 mM HEPES [(N-2-hydroxyethylpiperazine-N-2-ethane sulfonic acid)] buffer, 1% d-glucose, 5.0% sodium bicarbonate, gentamycin (40 μg/ml), and 10% human AB^+^ serum. Incubations were done at 37 °C and 5% CO_2_ level in a modular incubator. The culture was passaged with fresh mixture of erythrocytes and complete RPMI medium for every day to maintain cell growth. d-Sorbitol (5%) synchronised 1% ring stage parasitaemia in 5% HCT was used for the antimalarial assay using 96 well microtitre plate. A stock solution of 1.0 mg/ml of the test compound was prepared in DMSO and subsequent dilutions were made with incomplete RPMI to obtain different concentrations (50, 25, 12.5, 6.25, 3.13, 1.56, 0.78, 0.39, and 0.19 µg/ml) of samples (test/standard) in duplicate. In addition, drug free negative control to assess the parasite growth and chloroquine diphosphate as positive control to assess the integrity of the assay were also maintained in duplicate in the microtitre plate. After 40 h of incubation at 37 °C, the smears were prepared from each well (depending on the maturation of schizonts in negative control, ≥10%), stained with 3% Giemsa and examined under light microscope to ascertain drug sensitivity by assessing the level of parasitaemia, i.e. the inhibition of parasite growth in terms of percent inhibition of schizonts’ maturation^20^ ^,^ ^21^.

### Observation and assessment of activity

Each test compound was assayed in duplicate and number of schizonts was counted against 200 asexual parasites per replica under light microscope. The number of schizonts count was also assessed in negative control maintained in duplicate in the microtitre plate. Mean number of schizonts for duplicate observations was calculated at each concentration of test/standard samples compounds. Test values were compared with the control values. The percentage inhibition of parasite growth for each concentration was calculated as inhibition =100 – *A*, where *A* is the percentage inhibition of schizonts in test wells, which was determined using the following formula:

$$A=\frac{\text{Number of schizonts in the test well}}{\text{Number of schizonts in the control well}}\times 100$$

Further, the IC_50_ (concentration at which the inhibition of parasite growth represents 50%) values in µg/ml were also calculated using the NonLin *v*1.1 software^22^. Test results were compared with the standard results of CQ.

## *In silico* studies

### Molecular docking study

Molecular modelling studies were carried out using Dell Precision work station T3400 running Intel Core2 Duo Processor, 4 GB RAM, 250 GB hard disk and NVidia Quodro FX 4500 graphics card. Two-dimensional (2D) structures of all compounds were built on Chemdraw Ultra 10.0 (Cambridge Soft Co., Cambridge, MA, 2010) and Marvin Sketch (ChemAxon LLC, Cambridge, MA, 2015) software. The 2D structures were later transformed into 3D structures using the converter module of Biovia Discovery Studio (DS) *v* 4.5 (2015) software (San Diego, CA). The compounds were prepared using the prepare ligand module of the DS which includes assigning bond orders, generating various tautomers, ring conformations and stereochemistries. All the conformations generated were then energetically minimised by a single step Steepest Descent method using CHARMM (Chemistry at HARvard Macromolecular Mechanics, Cambridge, MA) Force Field with 5000 iterations and a minimum root mean square (RMS) gradient of 0.01 kcal/mol/Å^23^.

The X-ray crystal structure of falcipain 2-E-64 (PDB id: 3BPF) was retrieved from the RCSB Protein Data Bank (http://www.rcsb.org/pdb/) and Chain A of the protein determined at a resolution of 2.9 Å was used in the study^24^. Prior to docking, protein was prepared using protein preparation wizard tool of DS. Polar hydrogen atoms were added to the proteins and charges were assigned. All bound water molecules, other heteroatoms and ligands were excluded from the crystal structure as they were not significant for the proteins’ function. Subsequently, the 3D structure of protein was optimised by energy minimisation using CHARMM Force Field. It was done in two steps to remove the bad steric clashes using Steepest Descent and Conjugate Gradient methods for 5000 steps at RMS gradients of 0.01 and 0.05 kcal/mol/Å, respectively^23^.

Molecular docking studies were carried out using Biovia Discovery Studio (DS) *v* 4.5 (2015) software (San Diego, CA). After energy minimisation, the Chain A of falcipain 2 protein was defined as a receptor and the binding site sphere was selected based on the ligand binding location of E-64. A receptor grid was thereby generated around the binding cavity (active sites) of protein by specifying the key amino acid residues (Cys 42, Gly 83, and His 174)^24^. In DS, binding site sphere was set with a radius of 20 Å and *x*, *y*, *z* dimensions of –52.25, –4.46, –19.25, respectively. Flexible molecular docking was performed where the protein was held rigid while the ligands were allowed to be flexible during refinement. During docking, the falcipain 2-E-64 complex was imported and E-64 molecule (co-crystal ligand) was removed, and ligands were placed in the predicted binding site (grid box) and docking was performed using the Dock Ligands module of LibDock genetic algorithm program^25^ of DS. The docking parameters were as follows: No. of hotspots 100, docking tolerance 0.25, No. of runs 100, No. of iterations 1000, conformation method best, RMS gradient 0.01. All other docking and consequent scoring parameters used were kept at their default settings. The LibDock scores of the docked ligands were calculated. Different dock poses were studied to know the best binding mode of receptor–ligand complex in terms of scoring function. All docked poses were scored, ranked and the best pose of each compound having the highest score was selected and it was later used for the receptor–ligand interaction analysis. Interaction of ligands with receptor was studied to know the best binding orientation of receptor–ligand complex having maximum LibDock score. Binding modes of the best pose for each compound was also analysed with the help of 3D receptor–ligand complex. Different non-bonding interactions (hydrogen bonding and hydrophobic) were also analysed with the help of 2D diagram of docked receptor–ligand complexes. Receptor–ligand interaction gives better understanding of the molecular interactions between the binding site residues of receptor molecule and complimentary groups/atoms of ligands involved in interactions.

### Molecular properties calculation and drug-likeness studies

*In silico* calculations of the molecular properties and drug-likeness parameters for all compounds, **3a**–**e**, **3′a**–**n**, **3″a**–**e** were performed based on theoretical approaches to identify the compounds which violate the optimum requirements for drug-likeness. Molecular properties (molecular weight, Log*P* value, number of hydrogen bond acceptor(s) (HBA), number of hydrogen bond donor(s) (HBD), total polar surface area) incorporated in Lipinski’s rule of five^25^ and other physicochemical parameters like aqueous solubility (Log*S*), molar refractivity (MR), and molar volume (MV) were calculated using Calculation of Molecular Properties module of Biovia DS *v* 4.5 software (San Diego, CA). The number of rotatable bonds was predicted using Molsoft Online software (http://www.molsoft.com/, 2016) (San Diego, CA), and non-violation of drug-likeness and drug-likeness score was calculated using Molinspiration online software (http://www.molindpiration.com/, 2016) (Slovensky Grob, Slovakia).

### ADMET prediction

ADME-Toxicity (ADMET) for all the target compounds was calculated *in silico* using ADMET descriptor module of Biovia DS *v* 4.5 software (San Diego, CA). Six mathematical models (aqueous solubility, blood–brain barrier (BBB) penetration, cytochrome P450 2D6 inhibition, hepatotoxicity, human intestinal absorption, and plasma protein binding) were used to quantitatively predict properties related to ADMET characteristics or pharmacokinetics of molecules^26^. These properties influence oral bioavailability, cell permeation, and metabolism of drug molecules.

## Results and discussion

### Design of compounds

**Figure 2.**
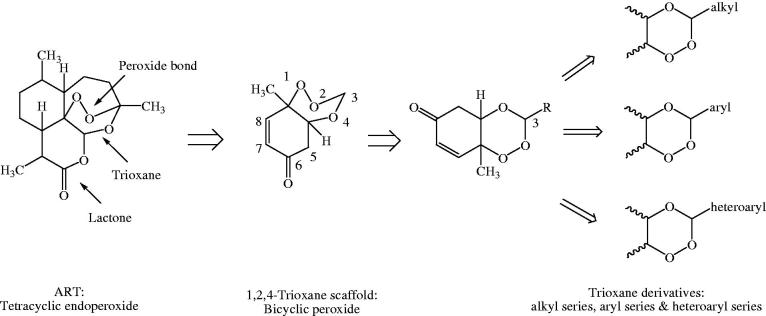
Design of 1,2,4-trioxane derivatives.

Rationale behind the design strategy involved selection of 1,2,4-trioxane ring system as the parent peroxide scaffold considering the pharmacodynamic importance of 1,2,4-trioxane component for antimalarial activity of ART and related endoperoxides. Our study was aimed at developing 1,2,4-trioxane derivatives as newer synthetic peroxide-based antimalarial agents having comparatively simpler molecular framework than conventional antimalarial drug molecules with activity against resistant malaria parasites. Three series of target compounds (Figure 2) were designed with diverse substitution patterns (alkyl/aryl/heteroaryl groups) at C-3 position of the 1,2,4-trioxane structural scaffold, based on the molecular manipulation approach of drug design with due consideration to all structure and property parameters that are relevant to biological activity. The 1,2,4-trioxane ring system was considered as the basic requirement for the antimalarial activity and substitutions with groups/moieties having electronic property of (s) was the main motif behind the design task.

### Chemistry

**Figure 3.**
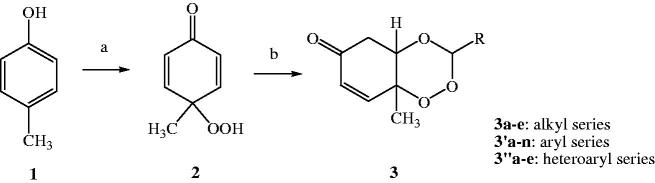
Scheme of synthesis of target compounds, **3a**–**e**, **3′a**–**n**, **3″a**–**e**. Reagents and conditions: (a) Oxone, NaHCO_3_/CH_3_CN/H_2_O, rt; (b) RCHO, 40–50 °C, 8–12 h, CH_2_Cl_2_, PTSA.

**Figure 4.**
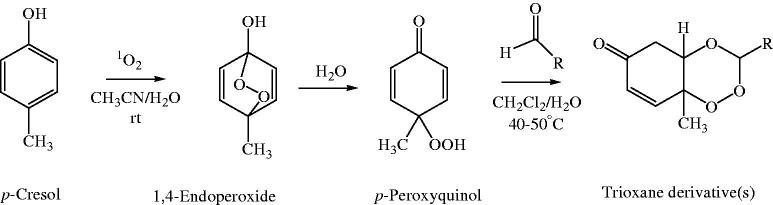
Reaction mechanism of synthesis of 1,2,4-trioxane derivative(s).

In our study, three new series of 1,2,4-trioxane derivatives were synthesised and evaluated *in vitro* for their antimalarial activity. Substitutions with different aliphatic, aromatic, and heteroaromatic groups at the C-3 position of 1,2,4-trioxane ring system afforded target trioxane derivatives obtained as single drug conjugates. The standard reaction procedure^11^ involving a facile synthetic route depicted in Figure 3 was employed for the preparation of 1,2,4-trioxane derivatives. The oxidative dearomatisation of *p*-cresol, **1** with oxone first yielded the intermediate compound, *p*-peroxyquinol, **2** which was later allowed to react with different aldehydes (aliphatic/aromatic/heteroaromatic) in the presence of an acid catalyst called PTSA, to obtain desired trioxane derivatives, **3a**–**e**, **3′a**–**n**, **3″a**–**e**. The first step of reaction was a [4 + 2] cycloaddition between electron rich *p*-alkyl phenol and 1O_2_ (singlet oxygen) generated *in situ* in the reaction system from oxone, which gave *p*-peroxyquinol intermediate through a 1,4-endoperoxide, i.e. peroxyhemiacetal. Subsequent desymmetrisation of *p*-peroxyquinol after reaction with aldehydes with the help of PTSA catalyst yielded different trioxane derivatives (Figure 4)^17^. The progress of reactions was monitored by the silica gel-G thin-layer chromatography (TLC) and the spots were visualised by iodine vapours. The purity of the synthesised compounds was ascertained by MP determinations and silica gel G TLC. All the compounds were obtained in good yields with high purity. The intermediate compound, 4-hydroperoxy-4-methyl-2,5-cyclohexadien-1one was obtained as white solid in 89% yield with melting range of 102–104 °C and *R*_f_ value of 7.8 (cyclohexane:EtOAc:acetic acid = 1:2:0.5). The physicochemical details of synthesised compounds are summarised in Table 1. The spectral (UV, IR, ^1^HNMR, ^13^CNMR, Mass) and analytical data (of representative compounds) depicted in the Experimental section are in close agreement with the structures of synthesised compounds. In UV spectra (in methanol), *λ*_max_ values were observed at the range of 212.8–344.0 which was due to the presence of chromophoric trioxane ring system along with alkyl/aryl/heteroaryl substituents at the C-3 position of the parent scaffold. IR spectral data showed absorption frequencies characteristic to specific functional groups and/or bonds present in the structure of synthesised compounds. The appearance of a broad peak at 3174.10 and 3269.19 cm^−1^ confirmed the presence of phenolic –OH group in the structure of **3′b** and **3′l**, respectively. N–H stretching band of 1*H*-pyrrol-2-yl and 1*H*-indol-3-yl systems was observed at 3247.01 and 3358.34 cm^−1^, respectively. The carbonyl (C=O) group was assigned with a strong and sharp absorption band in the region of 1694.10–1656.48 cm^−1^ for all the synthesised compounds. The characteristic peak due to N–O stretching (NO_2_) was also appeared for the compound **3′e** by a sharp peak of strong intensity at 1543.69 cm^−1^. A weak band at frequency of 1278.32 cm^−1^ was explicated due to the C–N stretch for compound **3′j**. Distinguished C–O stretching band appeared in the range of 1043.24–1177.34 cm^−1^. A peak of weak intensity observed at 1054.36 cm^−1^ was due to the aromatic C–Cl stretch for compound **3′d**. A weak absorption band observed at 892.28–820.17 cm^−1^ was due to O–O stretching. The presence of aliphatic C–H (CH_3_), aromatic C–H (CH=C), and aromatic C=C groups in the structure of synthesised compounds was also confirmed by characteristic peaks as depicted in the Experimental section.

**Table 1.** Physicochemical data of synthesised compounds.

Comp.	R	Colour (state)	% Yield	MP (°C)	*R*_f_^a^
**3a**	Ethyl	Colourless (solid)	78	112–114	0.70
**3b**	Propyl	Colourless (solid)	74	118–122	0.72
**3c**	Butyl	Brown (semi-solid)	69	–	0.74
**3d**	Pentyl	Brown (semi-solid)	66	–	0.74
**3e**	1-Formyl-but-4-yl	Brown (semi-solid)	65	–	0.67
**3′a**	Phenyl	Colourless (solid)	72	122–124	0.72
**3′b**	2-Hydroxyphenyl	Off-white (solid)	83	128–130	0.76
**3′c**	3-Methoxyphenyl	Pale yellow (solid)	74	145–147	0.72
**3′d**	4-Chlorophenyl	Colourless (solid)	84	136–140	0.74
**3′e**	4-Nitrophenyl	Yellow (solid)	80	156–158	0.72
**3′f**	4-Tolyl	Colourless (solid)	69	142–144	0.78
**3′g**	4-Bromophenyl	Brown (semi-solid)	68	–	0.68
**3′h**	4-Fluorophenyl	Brown (semi-solid)	66	–	0.68
**3′i**	1-Naphthyl	Brown (semi-solid)	70	–	0.70
**3′j**	4-Dimethylaminophenyl	Pale yellow (solid)	78	168–170	0.72
**3′k**	3-Cinnamyl	Yellow (solid)	65	–	0.66
**3′l**	4-Hydroxy-3-methoxyphenyl	Colourless (solid)	82	148–150	0.76
**3′m**	3,4-Dimethoxyphenyl	Colourless (solid)	76	128–130	0.74
**3′n**	Isophthalyl	Colourless (solid)	69	134–136	0.79
**3″a**	Furan-2-yl	Dark brown (semi-solid)	68	–	0.65
**3″b**	Thiophen-2-yl	Light brown (semi-solid)	66	–	0.66
**3″c**	Pyrrole-2-yl	Light yellow (solid)	64	145–147	0.67
**3″d**	Indole-3-yl	Colourless (solid)	75	157–160	0.74
**3″e**	Pyridin-4-yl	Brown (semi-solid)	68	–	0.68

a Cyclohexane:EtOAc:acetic acid (1:2:0.5).

^1^HNMR spectra exhibited resonance signals with chemical shift (*δ*, ppm) values characteristic to various structural protons which concorded the anticipated structure of synthesised compounds (Supplementary material). A singlet for three protons of CH_3_ group of the trioxane ring system was observed in the range of 1.16–1.34 ppm. For olefinic protons (CH**=**CH) peaks at 2.17–2.63 and 4.02–4.32 ppm were assigned, while for other skeletal protons like CO–CH_2_CH were assigned at 6.04–6.23 and 6.32–6.40 ppm. Depending on the nature of the substituent at C-3 position of the trioxane ring, distinct *δ* values were also observed with expected splitting patterns (doublets, triplets, or multiplets) and coupling constant (*J*) values. A broad singlet at 9.71 and 9.81 ppm was observed due to aromatic OH proton for compounds, **3′b** and **3′l**, respectively. The CHO proton in compound **3′h** was appeared at 9.71 ppm. Distinct singlet due to three protons of the OCH_3_ group appeared at 4.02 and 3.94 ppm for compounds **3′c** and **3′l**, respectively. Aromatic protons including heteroaryl substituents appeared in the range of 7.01–8.15 ppm. A broad singlet was also assigned to NH proton at 7.67 ppm and 9.87 ppm for compounds **3″c** and **3″d**, respectively. Aliphatic methyl and methylene protons were observed in multiplet pattern (doublet or triplet) at *δ* values in the downfield region for compounds **3a** and **3c**, as depicted in the Experimental section^28^.

In ^13^C NMR spectra, distinguished resonance signal due to the carbonyl (>C=O) carbon appeared in the range of 188.27–192.12 ppm. Aromatic/heteroaromatic carbons appeared at 107.23–162.25 ppm. Characteristic downfield signals for aliphatic carbons were observed for CH_3_ (14.10–15.65 ppm), CH_2_ (22.43–32.62 ppm), OCH_3_ (56.08 ppm, **3′l**; 29.12 ppm, **3′c**), and CH (36.36–92.68 ppm) groups. Olefinic (CH=CH) carbons were also assigned and depicted in the Experimental section. The mass spectra of **3a**–**e**, **3′a**–**n**, **3″a**–**e** exhibited prominent molecular ion peaks, [M]^+^ which finally confirmed the structure of compounds with the anticipated mass corresponding to their respective molecular formula. The results of elemental analyses were within the acceptable limits (±0.5%) of the calculated values for all the synthesised compounds.

### Antimalarial activity

**Figure 5.**
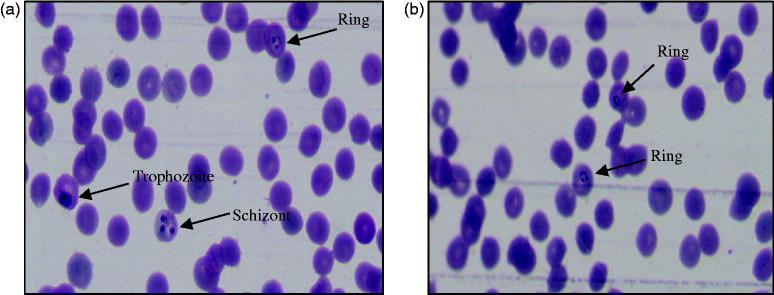
Photographs showing *in vitro* antimalarial activity of the most active compound, **3′l** against (a) CQ-sensitive 3D7 and (b) CQ-resistant RKL9 strains of *P. falciparum*.

The *in vitro* antimalarial activity data are summarised in Table 2. Results of *in vitro* antimalarial activity revealed that all the synthesised compounds (**3a**–**e**, **3′a**–**n**, **3″a**–**e**) showed activity against both CQ-sensitive (3D7) and CQ-resistant (RKL9) strains of *P. falciparum* in a dose dependant manner at screening doses. The IC_50_ values of tested compounds range from 1.24 µM to >1000 µM and 1.06 µM to >1000 µM against CQ-sensitive and CQ-resistant strains of *P. falciparum*, respectively. Out of 24 tested compounds, five compounds (**3d**, **3e**, **3′k**, **3″a**, and **3″b**) showed poor activity with IC_50_ values of >1000 µM against both the strains. Compounds **3a**–**3c**, **3′a**, **3′d**–**3′j**, **3′m**, **3′n**, and **3″c** exhibited moderate activity with IC_50_ values ranging from 18.37 to 220.20 µM and 14.72 to 220.68 µM against sensitive and resistant strains, respectively. Compounds, **3b**, **3c**, **3′a**, **3′e**, **3′h**, and **3′j** showed better activity against sensitive strain than the resistant one. Among three series of compounds, five compounds (**3′b**, **3′c**, **3′l**, **3″d**, and **3″e)** showed good activity, three compounds (**3′b**, **3′l**, and **3″d)** of which exhibited better activity against resistant strain than the sensitive one. These five compounds were found comparatively more potent than rest of the synthesised analogues. The IC_50_ values of two best compounds (**3′l** and **3″d)**, one (**3′l**) from aryl series and the other (**3″d**) from heteroaryl series were found to be 1.24 µM and 1.24 µM, respectively, against sensitive strain of *P. falciparum*. The IC_50_ values of the two compounds against resistant strain were found to be 1.06 µM and 1.17 µM, respectively. Results were comparable with that of the standard drug, CQ (IC_50_=1.23 µM and 47.16 µM against sensitive and resistant strain of *P. falciparum*, respectively). Figure 5 shows photographs of the effect of the most active compound, **3′l** against both CQ-sensitive (3D7) and CQ-resistant (RKL9) strains of *P. falciparum*.

**Table 2.** *In vitro* antimalarial activity data.

	IC_50_ in µg/ml (µM)^a^	
Comp.	*Pf* 3D7	*Pf* RKL9
**3a**	0.026 (26.20)	0.028 (28.46)
**3b**	0.020 (20.44)	0.022 (22.26)
**3c**	0.16 (160.47)	0.12 (120.13)
**3d**	3.48 (>1000)	4.09 (>1000)
**3e**	3.46 (>1000)	4.07 (>1000)
**3′a**	0.068 (68.46)	0.066 (66.19)
**3′b**	0.0054 (5.43)	0.0056 (5.68)
**3′c**	0.0074 (7.62)	0.0078 (7.83)
**3′d**	0.032 (32.49)	0.032 (32.54)
**3′e**	0.040 (40.78)	0.036 (36.42)
**3′f**	0.052 (52.06)	0.054 (54.17)
**3′g**	0.058 (58.20)	0.058 (58.34)
**3′h**	0.040 (40.21)	0.036 (36.67)
**3′i**	0.074 (74.22)	0.078 (78.28)
**3′j**	0.018 (18.37)	0.014 (14.72)
**3′k**	1.20 (>1000)	1.48 (>1000)
**3′l**	0.0012 (1.24)	0.0010 (1.06)
**3′m**	0.056 (56.09)	0.064 (64.53)
**3′n**	0.045 (45.29)	0.048 (48.20)
**3″a**	2.07 (>1000)	5.02 (>1000)
**3″b**	3.50 (>1000)	3.61 (>1000)
**3″c**	0.22 (220.20)	0.22 (220.68)
**3″d**	0.0012 (1.24)	0.0011 (1.17)
**3″e**	0.0072 (8.27)	0.0065 (6.52)
**CQ**	0.0012 (1.23)	0.147 (47.16)

a Calculated using *NonLin v1.1* software, mean of two replicate observations (counted against 200 cells per replicate), values in parentheses indicate activity in micromolar (µM) concentration.

**Figure 6.**
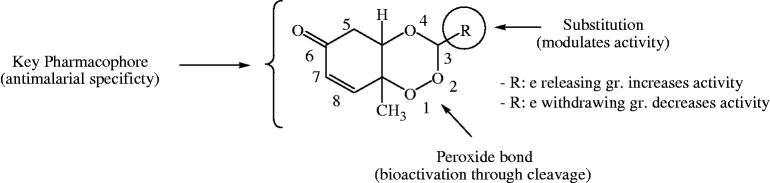
Structural requirements and effect of substitutions on antimalarial activity of 1,2,4-trioxane derivatives.

Results reveal that the degree of antimalarial (inhibitory) activity of 1,2,4-trioxane derivatives is dependent on the nature and degree of substitution pattern. Different structural substitutions such as non-bulky alkyl and bulky aryl/heteroaryl groups at C-3 position of the ring system modulate the antimalarial efficacy of prepared 1,2,4-trioxane derivatives. Some degree of variations in activity among synthesised analogues might be due to diverse structural substitutions. A brief SAR study can be depicted as follows: small alkyl substituents like ethyl, propyl, or butyl moieties are of considerable importance for the activity, while substitution with larger alkyl like pentyl and 1-formyl-but-4-yl moieties is comparatively less important for the activity. Substitution with bulky aryl moieties except 3-cinnamyl group also contributes good antimalarial potency to same extent or little less or even more compared to alkyl substituted analogues. Compounds with phenyl, 4-chlorophenyl, 4-nitrophenyl, 4-tolyl, 3,4-dimethoxyphenyl, and isophthalyl substitutions sufficiently retain the activity, whereas, in case of 2-hydroxyphenyl, 3-methoxyphenyl, 4-hydroxy3-methoxyphenyl substituted analogues the activity significantly increases. With bulky heteroaryl moieties like furan-2-yl and thiophen-2-yl substitutions, the activity declines, while, pyrrole-2-yl and pyridine-4-yl improve activity, and the indole-3-yl substitution still potentiates the activity. It is also clear that compounds with five membered heterocyclic rings are less important for the activity than six membered or fused heteroaryl systems. Upon critical analysis of SAR, the most notable point found is that substitutions with electron withdrawing moieties are likely less important for the activity, while electron releasing groups substituents are considerably more important for activity. More illustratively, phenyl substituent with electron releasing (OH, OCH_3_, etc.) groups significantly increases the activity, whereas, electron-withdrawing group (Cl, NO_2_, etc.) substituted phenyl ring reduces the activity (Figure 6). Substitutions of electron releasing groups at *ortho* and *para* or both have greater contributing effects than *meta* substitutions. Di-substitution (4-hydroxy-3-methoxyphenyl, compound **3′l**) is more important for the activity than mono-substitution (2-hydroxyphenyl, compound **3′b**). The findings of our present study are also consistent with earlier report^28^ on trioxane-based compounds which show excellent antiparasitic activity upon incorporation of an aryl group. It is seen that bulkiness of substituents is important for the activity. However, aryl substitution seems to be more important than alkyl or heteroaryl substitution. It is important to note here that besides bulkiness of substituents, lipophilicity (hydrophobicity) and ionisation potential (basicity) of the molecule are also considered to play a crucial role for the activity. The lipophilicity (Log*P*) is inevitable for permeation of parasitic membrane and basicity (pKa) is required for accumulation of trioxane molecule in the acidic food vacuole of parasites.

**Figure 7.**
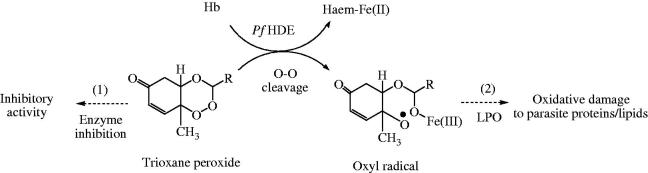
Hypothetical mode(s) of antimalarial action of 1,2,4-trioxane derivatives. (1) Inhibition of haemoglobin degrading enzyme in food vacuole of parasite, (2) direct lethal action to parasite by LPO process. *Pf* HDE: *P. falciparum* haemoglobin degrading enzyme; LPO: lipid peroxidation.

Literature report that 1,2,4-trioxane structure lacking the lactone ring plays an important role for the antimalarial activity, but 1,2,4-trioxane ring alone is insufficient for optimal antimalarial potency as compared to ART and its semi-synthetic analogues^29^. The reductive cleavage of the peroxide bond *in vivo* by heme [Fe(II)] (released during haemoglobin degradation process in parasitised RBC) and subsequent production of cytotoxic carbon-centred radicals target cell proteins/enzymes and destroy parasites^30–32^. From the SAR study, possible mode(s) of antimalarial action (Figure 7) of 1,2,4-trioxane derivatives can therefore be explained as: target compounds may act either as direct inhibitor of enzyme (receptor antagonist) or as prodrug by interfering the haemoglobin degradation mechanism during erythrocytic stages of *P. falciparum*. Electronic property of the substituent may play a significant role for the bio-activation (through O–O cleavage) of trioxane ring system to generate highly reactive oxyl radical which causes death of parasite by lipid peroxidation of parasitic cell membrane/protein.

### Docking simulations

*Plasmodium* cysteine protease falcipain 2 enzyme plays an essential role in the degradation of host haemoglobin into smaller peptides which takes place in the acidic food vacuole of *P. falciparum.* Recent literature^33^ claims that endoperoxide antimalarials have potential to inhibit falcipain 2 enzyme and therefore, drug targeting of this particular enzyme would be an attractive avenue in the design and development of new antimalarial drugs. Accordingly, a molecular docking study was performed for all newly designed compounds **3a**–**e**, **3′a**–**n**, **3″a**–**e** using the falcipain 2 enzyme as receptor molecule.

**Figure 8.**
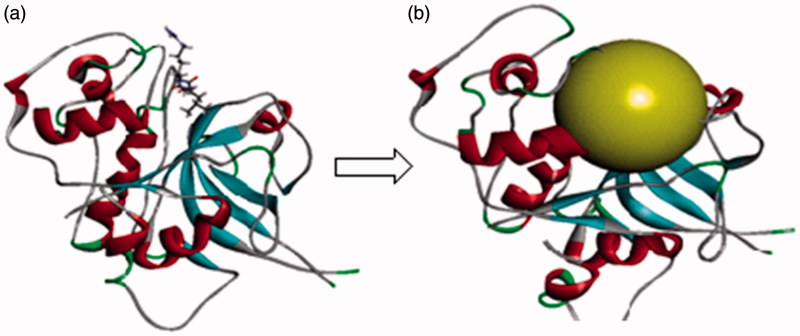
(a) Optimised co-crystal structure of falcipain 2 (chain A)-E-64 and (b) receptor grid for docking.

**Figure 9.**
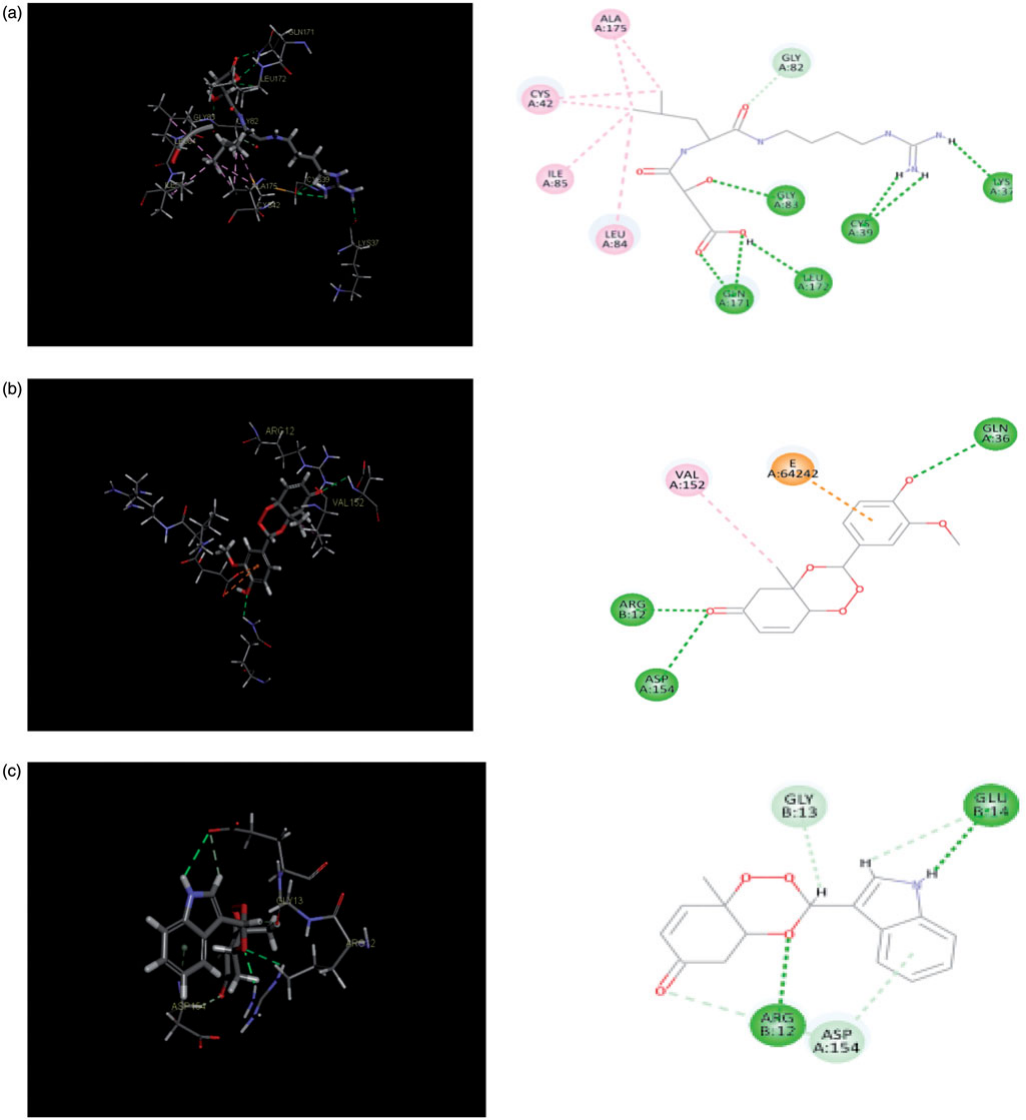
(a) Redocked conformer (pose) of E-64 in the active site of the protein falcipain 2 (left) and 2D representation of the binding interaction (right); (b, c) binding mode (left) and 2D receptor–ligand interaction diagram (right) of compounds, **3′l** and **3″d** at binding pocket of falcipain 2 (left), respectively.

The protein model used for docking study was validated as follows: the 3D crystal structure of falcipain 2 co-crystallised with the inhibitor *trans*-epoxysuccinyl-*L*-leucylamido-(4-guanidino) butane (E-64) with active site (receptor grid model) as defined by Cys 42, Gln 36, and His 174 residues was optimised and used for the study. The co-crystal structure of falcipain 2-E-64 (PDB: 3BPF) and the receptor grid model used for docking study are represented in Figure 8. Co-crystallised ligand, E-64 was re-docked using flexible docking simulations (LibDock program of DS) into the original structure of the target protein, falcipain-2 by non-covalent docking procedure. For this study, docking parameters were set to the software’s default values. E-64 was successfully re-docked to the predicted active sites of falcipain 2 with an acceptable RMSD value of 1.124 Å. Further, in order to reproduce an experimentally observed ligand-binding mode, the co-crystallised ligand, E-64 (a selective falcipain 2 inhibitor) was used as reference ligand. Results confirmed experimental binding conformations of E-64 in the binding pocket of receptor molecule. The binding modes and ligand–protein interaction diagrams obtained in re-docking of E-64 are shown in Figure 9(a).

Docking results revealed that LibDock program successfully docked all 1,2,4-trioxane derivatives into the binding pocket of falcipain 2 enzyme. All compounds which were active in *in vitro* test could bind with the active site of falcipain-2 with high docking score (LibDock) and binding affinities in the range of 96.077–126.390. The LibDock scores are summarised in Table 3. All trioxane derivatives except **3a** and **3b** exhibited more binding affinity (higher dock scores) than the co-crystal ligand, E-64 (LibDock score =100.364). Five compounds which showed most potency in *in vitro* study also ranked top in docking study with LibDock scores of 116.533, 126.390, 122.609, 110.646, and 124.737 for **3′a**, **3′b**, **3′l**, **3′d**, and **3′e**, respectively. It is important to note here that docking results are generally analysed by a statistical scoring function which converts interacting energy (binding energy) into numerical values called as the docking score, and therefore docking score is nothing but an expression of binding energy^34^. Binding energy (Δ*G*) includes the sum of all non-bonded interactions including hydrogen bonding, hydrophobic and Vander Walls between protein residues and bound ligand^35^. LibDock score is useful to assess the antimalarial activity of ligands as *P. falciparum* falcipain 2 inhibitors. Docking scores of compounds supported the results of *in vitro* antimalarial activity of synthesised 1,2,4-trioxane derivatives.

**Table 3.** LibDock scores, no. of H-bonds and H-bond energies.

Comp.	LibDock score	No. of H-bond (s)	H-Bond energy
**3a**	93.077	3	−2.056
**3b**	96.153	3	−1.648
**3c**	101.69	3	−2.174
**3d**	111.343	3	−2.056
**3e**	108.143	5	−5.457
**3′a**	107.807	4	0.804
**3′b**	116.533	6	−3.727
**3′c**	126.390	4	−2.265
**3′d**	110.908	3	0.179
**3′e**	127.665	4	−2.246
**3′f**	118.391	5	0.173
**3′g**	117.436	2	0.182
**3′h**	110.983	3	0.174
**3′i**	120.229	3	0
**3′j**	118.985	4	−2.5
**3′k**	120.370	1	−2.526
**3′l**	122.609	3	0.976
**3′m**	100.364	1	−2.526
**3′n**	122.864	4	0.780
**3″a**	101.911	3	−2.5
**3″b**	105.097	5	−2.5
**3″c**	110.952	6	−2.466
**3″d**	110.646	5	−2.478
**3″e**	124.747	6	0.031
**E-64**	100.364	7	0.086

Protein–ligand docking was performed to generate the bioactive binding poses of designed inhibitors in the active site of falcipain 2 enzyme. LibDock is a high throughput docking algorithm that finds various conformations of the ligands in the protein active site based on polar interaction sites (hotspots)^36^. The 3D poses of bound ligands were visualised which revealed that the best orientation of the ligand relative to the receptor as well as the conformation of the ligand and receptor (best fit of ligand in the receptor molecule). Analysis of 2D diagram indicated that various non-bonded interactions mainly polar hydrogen bonding interactions were involved between binding site residues (active site amino acids) and ligand moieties/atoms. Table 3 also depicts the number of hydrogen bonds (H-bonds) and H-bond energies for all docked compounds. Higher the number of hydrogen bonds, higher is the binding affinity. Apart from hydrogen bonding interactions, other non-bonded interactions like hydrophobic bonding were also observed.

Table 4 reveals the hydrogen bonding interactions of five most potent compounds (**3′b**, **3′c**, **3′l**, **3″d**, and **3″e)** with active site residues such as Asp 154, Arg 12, Gln 36, Glu 14, Glu 15, Glu 138, Gly 13, Val 152, and His 19. Compounds **3′l** and **3″d** which showed the highest antimalarial activity among three series, could bind the active sites of falcipain-2 mainly by hydrogen bonding interactions. The 3D binding modes and 2D interaction diagrams of two most potent compounds, **3′l** and **3″d** are shown in Figure 9(b,c), respectively. The first compound formed three strong hydrogen bonds with residues like Gln 36 (O··H··O), Asp 154 (O··H··N), and Arg 12 (O··H··O) with bonding distances of 2.737, 2.985, and 1.834 Å, respectively. In later case, five bonds were observed with residues like Arg 12 (O··H··O), His 19 (O··H··H), Gly 13 (O··H··O), Glu 14 (O··H··O), and Glu 15 (O··H··O) with bonding distances of 1.868, 2.938, 2.308, 2.795, and 2.489 Å, respectively. Analysis of best docking poses of **3′l** and **3″d** revealed that the trioxane scaffold was oriented in the binding cavity (active site residues) of falcipain 2 receptor molecule. In 2D diagram, the trioxane moiety could occupy the binding sites of falcipain-2 through strong H-bonding interactions along with hydrophobic interactions. Such interactions afforded good stability between receptor molecule and ligand. Trioxane moiety interacted with multiple amino acid residues such as Arg 12, Asp 154, Val 12 and Gly 13, and Gln 36 and Glu 14 residues interacted with the 4-hydroxy-3-methoxyphenyl and idole-3-yl substituents in compounds **3′l** and **3″d**, respectively. Strong H-bonding interactions between C=O group of the trioxane and amino acid residues were also observed. The OH and NH groups were involved for H-bonding interactions from the substituent moieties for compounds **3′l** and **3″d**, respectively. On further analysis of docking interactions, it was found that trioxane ring played a crucial role in protein–ligand binding. Substituents increased binding strength by forming additional H-bonds that facilitated much stronger interaction of ligands with the receptor (falcipain-2 protein) molecule with optimum binding affinity to achieve desired antimalarial activity.

**Table 4.** Details of hydrogen bonding between five most active ligands and receptor molecule.

		H-binding ligand		H-binding receptor			
Comp.	H-bond (s)	Element	Type	Residue	Element	Type	H-bond distance (Å)
**3′b**	6	O	A	Asp 154	N	D	2.749
		O	A	His 19	H	D	2.811
		O	A	Arg 12	H	D	1.720
		O	A	Arg 12	H	D	2.393
		H	D	Gly 13	O	A	2.488
		H	D	Val 152	O	A	2.262
**3′c**	4	O	A	Asp 154	N	D	2.234
		O	A	His 19	H	D	2.534
		H	D	Asp 154	O	A	2.874
		H	D	Glu 138	O	A	3.016
**3′l**	3	O	A	Gln 36	H	D	2.737
		O	A	Asp 154	N	D	2.985
		O	A	Arg 12	H	D	1.834
**3″d**	5	O	A	Arg 12	H	D	1.868
		O	A	His 19	H	D	2.938
		H	D	Gly 13	O	A	2.308
		H	D	Glu 15	O	A	2.795
		H	D	Glu 14	O	A	2.489
**3″e**	6	O	A	Arg 12	H	D	2.976
		O	A	Arg 12	H	D	2.107
		H	D	Glu 14	O	A	2.804
		O	A	Asp 154	H	D	2.515
		H	D	Gly 13	O	A	2.247
		H	D	Glu 14	O	A	2.735

Docking is generally used to find the best binding orientation of small molecules bound to their target protein molecules in order to predict the binding affinity and thereby biological activity of the small molecules. Hence, docking plays an important role in the rational drug design. To evaluate the ligand–receptor interactions, the best pose of receptor–ligand complex should possess the lowest free energy (binding energy) which differs from experimentally observed other structure and estimate the binding affinity. Search algorithm is used to generate different poses of the ligand within the active site of molecules, orientations of particular conformations of the molecule in the binding site. Scoring function is used to identify the most likely pose for an individual ligand to assign a priority order to a set of diverse ligands docked to the same protein and hence estimate binding affinity. Using scoring function, one can predict the binding conformation of a ligand in its receptor and the affinity between the ligand and the receptor with the correct poses of ligands in the binding pocket of a protein^34–38^.

**Figure 10.**
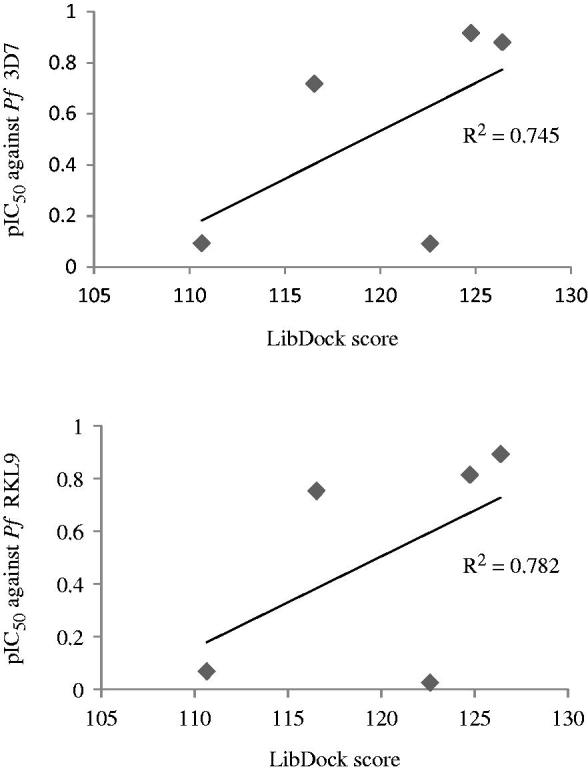
Graphs showing correlation between *in vitro* antimalarial activity (pIC_50_) and LibDock scores for five most potent compounds.

Furthermore, a correlation study between docking scores and logIC_50_ (pIC_50_) values of top five compounds (**3′b**, **3′c**, **3′l**, **3″d**, and **3″e**) was carried out. Graphs presented in Figure 10 revealed that a good correlation existed between *in vitro* antimalarial activity (against both CQ-sensitive and CQ-resistant *P. falciparum* strains) of compounds and their LibDock scores. The *in vitro* activity was found consistent (*R* ^2^ = 0.745 or 0.782) with the binding affinity of compounds for the *P. falciparum* cysteine protease falcipain 2 receptor.

### Molecular properties and drug-likeness

The results of predicted Lipinski’s parameters and other drug-likeness properties of the synthesised compounds, **3a**–**e**, **3′a**–**n**, **3″a**–**e** are depicted in Table 5. Results revealed that all the compounds possessed good drug-like properties based on Lipinski’s rule of five with additional parameters such as molar solubility (MS), MV, MR, and number of rotatable bonds (nRotB). All compounds obeyed Lipinski’s rule of five and Veber rule. Lipinski rule of five is a rule to evaluate drug likeness to determine if a chemical compound has a certain pharmacological or biological activity to make it an orally active drug^39^. According to Lipinski’s rule, compounds are more likely to be drug-like and orally bioavailable if they obey the following criteria: Log*P*_o/w_ (octanol/water partition coefficient) ≤ 5, MW (molecular weight) ≤ 500, HBA ≤ 10, and HBD ≤5^15^. To further substantiate, Veber et al. stated that compounds with ≤10 rotatable bonds and TPSA (total polar surface area) of ≤140 A^2^ are more likely to show membrane permeability and good bioavailability^39^ ^,^ ^40^. Lipinski rule of five is considered predictive for oral bioavailability; however, 16% of oral drugs violate at least one of the criteria and 6% fail in two or more^39^. In our study, compounds did not violate Lipinski rule of five parameters. Poor absorption or permeation of a ligand is more likely if a drug-like molecule have more than one of five rule violations. Values of Log*P*, MW, and TPSA indicated that compounds possessed good membrane permeability and oral bioavailability, whereas, nRotb bonds suggested that compounds had good intestinal availability. MS data indicated good bioavailability of compounds if given by oral route. MV and MR values were also found in permissible range which indicated good oral bioavailability for all the compounds. Hydrophobicity, membrane permeability, and bioavailability are dependent on molecule’s MW, Log*P*, MS, HBA, and HBD. Molecules violating more than one of these rules fail to exhibit optimum bioavailability. Sufficient water solubility is also important for optimal bioavailability of drugs. Number of rotatable bonds is important for molecular conformational studies (i.e. stereoselectivity of drug molecules) for optimal binding with the receptor molecule. Reduced molecular flexibility, as measured by the number of rotatable bonds, and polar surface area or total hydrogen bond count (sum of donors and acceptors) are some important predictors of good oral bioavailability, independent of molecular weight^15^. Further, TPSA, MV, and MR are also useful parameters for drug’s transport and biodistribution^15^. The drug score combines drug-likeness, lipophilicity, solubility, molecular weight, and the risk of toxicity into a single numerical value that can be used to predict a global value for each compound as a potential new drug candidate^22^. Drug-likeness score was obtained in the range of −0.18 and −0.79. Even though compounds exhibited negative drug-likeness values, scores were in acceptable range and indicated that compounds possessed potential as new drug candidates. The overall analysis of drug-likeness studies strongly suggested that newly designed 1,2,4-trioxane derivatives possessed good drug-likeness behaviour favourable for optimal membrane permeability, transport and bioavailability and eventual interaction with the receptor molecule.

**Table 5.** Calculated molecular properties and drug-likeness parameters.

	Lipinski’s parameters										
Comp.	MW	Log*P*	nHBA	nHBD	TPSA (A^2^)	nViolations	MS	MR	MV (A^3^)	nRotB	DL score
**3a**	198.78	1.02	4	0	44.76	0	−1.33	50.01	220.52	1	−0.43
**3b**	212.25	1.50	4	0	44.76	0	−1.74	54.65	238.37	2	−0.41
**3c**	226.27	1.99	4	0	44.76	0	−2.21	59.25	256.28	3	−0.60
**3d**	240.30	1.64	4	0	44.76	0	−2.23	59.68	258.69	4	−0.68
**3e**	240.26	0.69	5	0	61.83	0	−1.81	59.99	264.63	4	−0.42
**3′a**	246.26	2.17	4	0	44.76	0	−2.68	65.27	255.79	1	−0.82
**3′b**	262.26	1.79	5	1	64.99	0	−2.21	66.97	268.63	1	−0.17
**3′c**	276.29	2.26	5	0	53.99	0	−2.81	71.74	287.64	2	−0.54
**3′d**	280.71	2.88	4	0	44.76	0	−3.48	70.08	272.98	1	−0.26
**3′e**	291.26	1.84	6	0	90.58	0	−3.19	72.60	280.74	2	−1.06
**3′f**	260.29	2.57	4	0	44.76	0	−3.19	70.32	276.73	1	−0.78
**3′g**	325.16	3.02	4	0	44.76	0	−3.85	72.90	277.64	1	−0.65
**3′h**	264.25	2.44	4	0	44.76	0	−2.98	65.49	261.70	1	−0.45
**3′i**	296.32	3.50	4	0	44.76	0	−4.39	81.73	306.92	1	−0.76
**3′j**	289.33	2.29	5	0	48.00	0	−2.64	79.70	305.35	2	−0.79
**3′k**	272.30	3.01	4	0	44.76	0	−3.66	75.59	296.66	2	−0.70
**3′l**	292.29	1.88	6	1	74.22	0	−2.32	73.43	299.06	2	−0.18
**3′m**	306.32	2.23	6	0	63.22	0	−2.89	78.21	319.92	3	−0.10
**3′n**	274.27	1.96	5	0	61.83	0	−2.21	62.76	284.05	2	−0.72
**3″a**	236.23	1.32	4	0	57.90	0	−2.08	57.68	241.85	1	−0.70
**3″b**	252.28	1.76	4	0	73.00	0	−2.68	64.13	251.15	1	−0.51
**3″c**	235.24	0.99	4	1	60.55	0	−1.63	59.93	243.60	1	−1.22
**3″d**	285.30	1.12	5	0	57.65	0	−3.25	76.36	251.09	1	−0.39
**3″e**	247.25	2.39	4	1	60.55	0	−2.21	71.87	296.39	1	−1.24

MW: molecular weight; Log*P*: log of octanol/water partition coefficient; nHBA: no. of hydrogen bond acceptor(s); nHBD: no. of hydrogen bond donor(s); TPSA: total polar surface area; nViolations: no. of rule of five violations; MS: molar aqueous solubility; MR: molar refractivity; MV: molar volume; nRotB: no. of rotatable bonds; DL: drug-likeness.

Since all newly designed compounds showed favourable drug-like properties, a reasonable correlation can be drawn between their calculated drug-like properties and *in vitro* antimalarial activity profile. Log*P*_o/w_ is a direct indicator of lipophilicity of drug substances. Higher the value of Log*P*, better the biological membrane permeability. It is inevitable for a molecule to have sufficient lipophilicity which is required for its optimal bioavailability and biological action. Hydrogen bond acceptor and donor groups are of paramount importance required to achieve optimal drug action. PSA is also closely related to the hydrogen bonding potential of a drug molecule. Practically, a compound with all drug-like properties in the desired range appears to exhibit high levels of therapeutic potency. Good drug-like properties and activity are complementary, and hence balanced attention to both properties and bioactivity could suitably transform a ligand to a good drug lead^41–44^. Better antimalarial activity of the top five compounds **3′b**, **3′c**, **3′l**, **3″d**, and **3″e** might be probably due to their sufficient lipophilicity along with balanced polar properties likes HBD, HBA groups, rotatable bonds, and PSA. They collectively determine membrane permeability as well as transport property of drug molecule. Due to optimal lipophilicity, compound readily penetrated parasite’s cell membrane that led to attain an intracellular concentration for desired antimalarial activity.

### ADMET prediction

The ADMET values of newly designed 1,2,4-trioxane derivatives presented in Table 6 were found in acceptable range with favourable ADMET properties. All the compounds were predicted to have good intestinal absorption and non-inhibitors of cytochrome P450 2D6 (CYP2D6) with medium to moderate BBB penetration. BBB penetration is mandatory for the drug to be used in the treatment of cerebral malaria. The CYP2D6 enzyme is one of the important enzymes involved in drug metabolism. The aqueous solubility prediction (defined in water at 25 °C) indicated that most of the compounds were soluble in water. The predictive hepatotoxicity was observed for a few compounds among three series. Some of the compounds were found to be highly bound with plasma protein, while some were poorly bound with plasma protein.

**Table 6.** Theoretical ADMET parameters.

Comp.	Aqueous solubility	BBB penetration	CYP P450 2D6 inhibition	Hepatotoxicity	Intestinal absorption	PP binding
**3a**	3	2	FALSE	TRUE	0	FALSE
**3b**	3	2	FALSE	FALSE	0	FALSE
**3c**	3	1	FALSE	FALSE	0	FALSE
**3d**	3	3	FALSE	FALSE	0	FALSE
**3e**	3	3	FALSE	FALSE	0	FALSE
**3′a**	2	1	FALSE	FALSE	0	TRUE
**3′b**	3	2	FALSE	FALSE	0	TRUE
**3′c**	2	2	FALSE	FALSE	0	TRUE
**3′d**	2	1	FALSE	FALSE	0	TRUE
**3′e**	2	3	FALSE	TRUE	0	TRUE
**3′f**	2	1	FALSE	FALSE	0	TRUE
**3′g**	2	1	FALSE	FALSE	0	TRUE
**3′h**	2	1	FALSE	TRUE	0	TRUE
**3′i**	2	1	FALSE	TRUE	0	TRUE
**3′j**	2	1	FALSE	TRUE	0	TRUE
**3′k**	2	1	FALSE	FALSE	0	TRUE
**3′l**	3	2	FALSE	FALSE	0	FALSE
**3′m**	2	2	FALSE	FALSE	0	TRUE
**3′n**	2	2	FALSE	FALSE	0	TRUE
**3″a**	3	2	FALSE	TRUE	0	TRUE
**3″b**	2	2	FALSE	TRUE	0	TRUE
**3″c**	3	2	FALSE	FALSE	0	FALSE
**3″d**	3	2	FALSE	FALSE	0	FALSE
**3″e**	2	2	FALSE	TRUE	0	FALSE

Aqueous solubility: 3, Good, 2, Low; BBB (blood–brain barrier) penetration: 3, Low, 2, Medium, 1, Moderate; Cytochrome (CYP) P450 2D6 inhibition: false-non-inhibitor; hepatotoxicity: true-toxic, false-non-toxic; intestinal absorption: 0, Good; plasma protein (PP) binding: true-highly bounded, false-poorly bounded.

## Conclusions

Newer series of 1,2,4-trioxane derivatives reported herein have potential *in vitro* antimalarial effectiveness and therefore, further studies are required for the evaluation of their toxicity, antimalarial efficacy, and pharmacokinetics in animal models. Our work is in progress to carry out *in vivo* toxicity and antimalarial activity of potent compounds. Structure–activity relationship study helped us to understand the importance of the core 1,2,4-trioxane ring system and also the role of substitution pattern on the overall antimalarial activity of newly designed trioxane analogues. Pharmacodynamic significance of the basic structural scaffold and unique structural simplicity of trioxane molecule highlighted the potential of 1,2,4-trioxane derivatives to be used as future antimalarial agents. *In silico* studies confirmed that newer 1,2,4-trioxane derivatives developed as single drug conjugates possessed target specificity for the *P. falciparum* cysteine protease falcipain 2 receptor with well-defined physicochemical, pharmacokinetics, and toxicity properties. All these results yielded valuable information for further optimisation of 1,2,4-trioxane-based compounds as falcipain 2 inhibitors based on the structure-based drug design approach. Further QSAR and pharmacokinetic-based optimisation studies are therefore required to develop antimalarial drug candidates as novel falcipain 2 inhibitors from our present 1,2,4-trioxane lead molecules, particularly 4-hydroxy-3-methoxysubstituted or indole substituted compounds.

## Supplementary Material

Supplementary materials
