# Supplementary material for: Novel series of 1,2,4-trioxane derivatives as antimalarial agents
Source: J Enzyme Inhib Med Chem. 2017 Sep 5;32(1):1159–73. doi: 10.1080/14756366.2017.1363742 (PMC6009891; doi:10.1080/14756366.2017.1363742)
Supplement: Supplementary materials [file IENZ_A_1363742_SM3028.pdf]

SUPPLEMENTARY MATERIALS

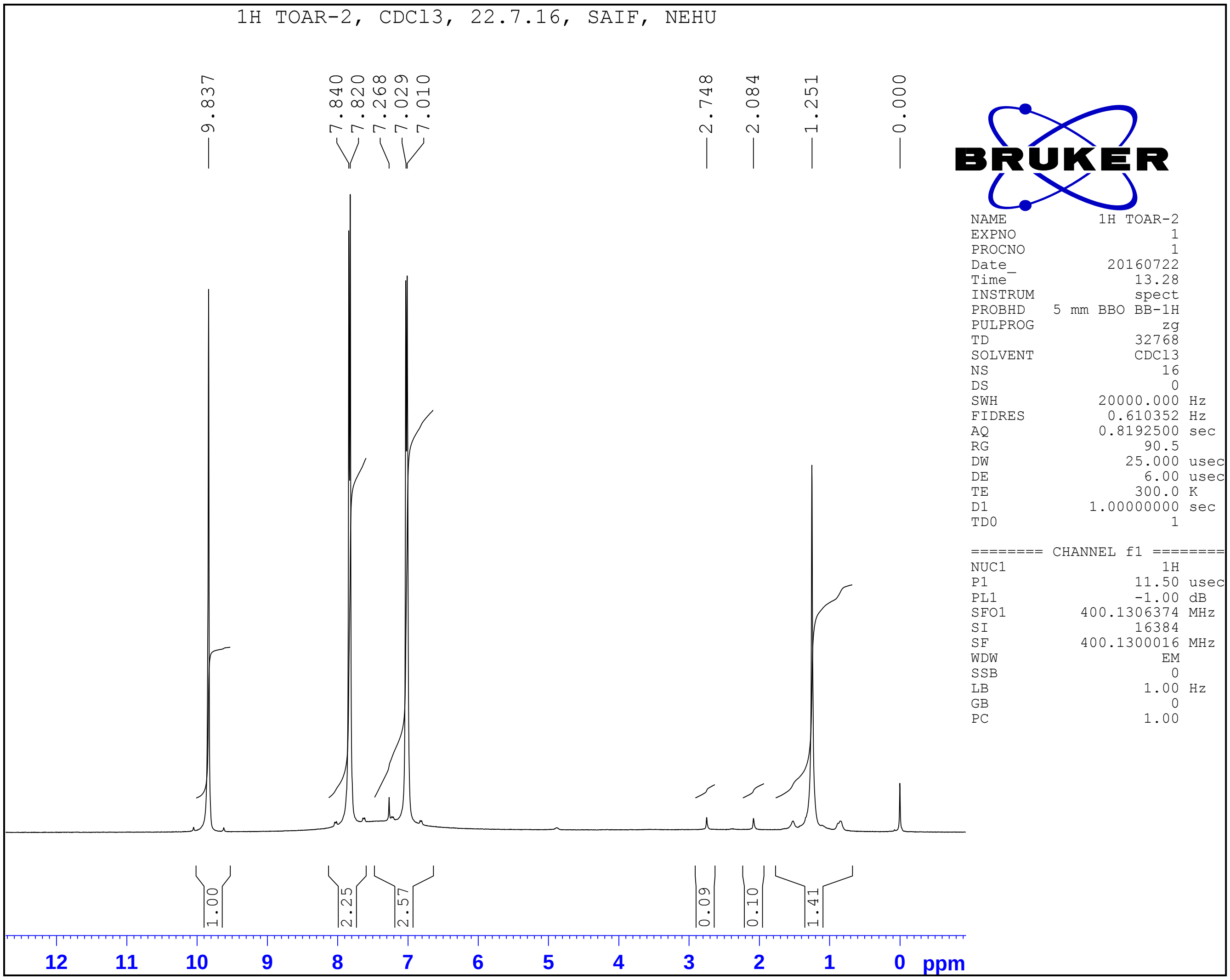

^{1}H NMR spectrum of compound **3'b**

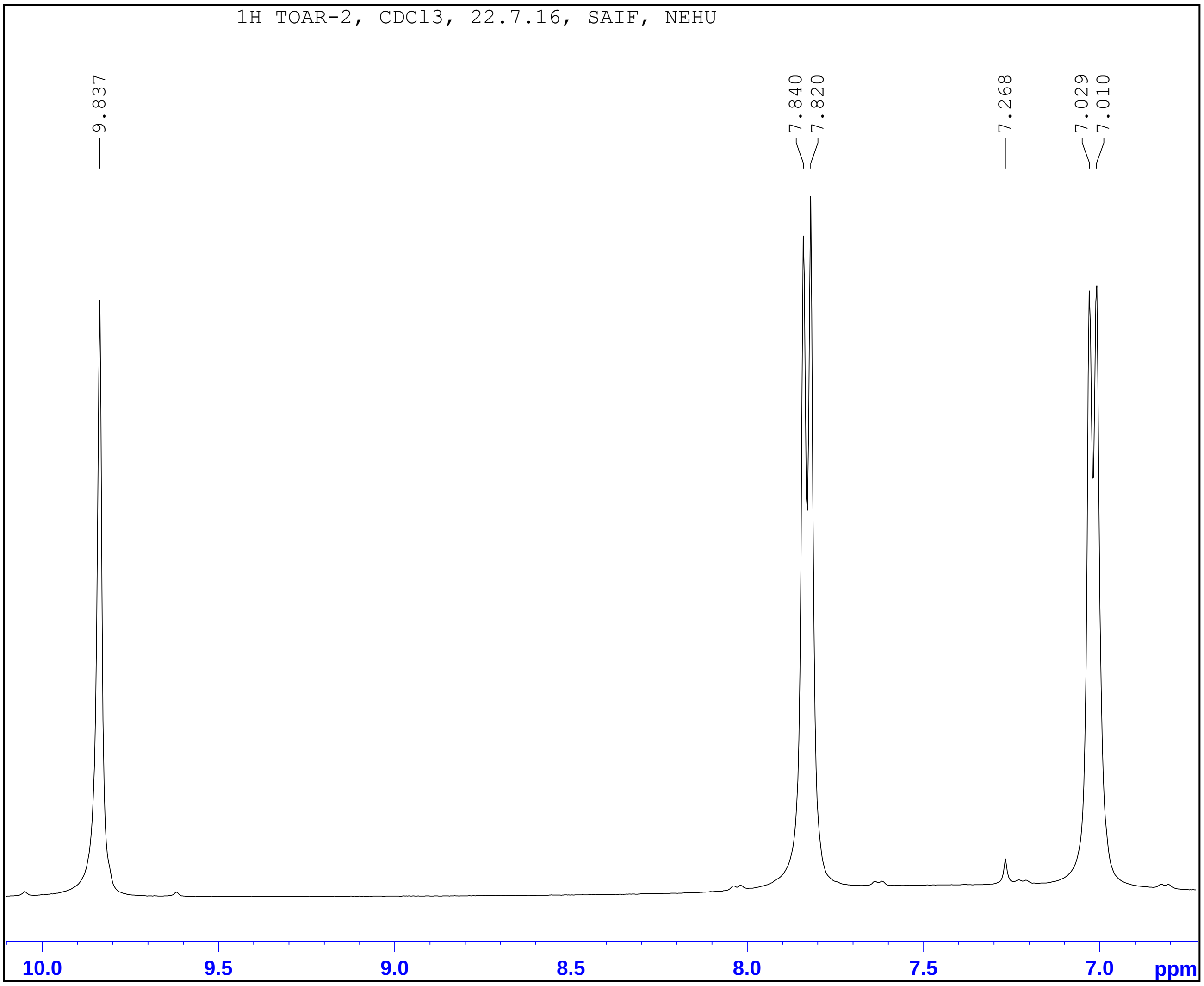

^{1}H NMR spectrum of compound **3'b**

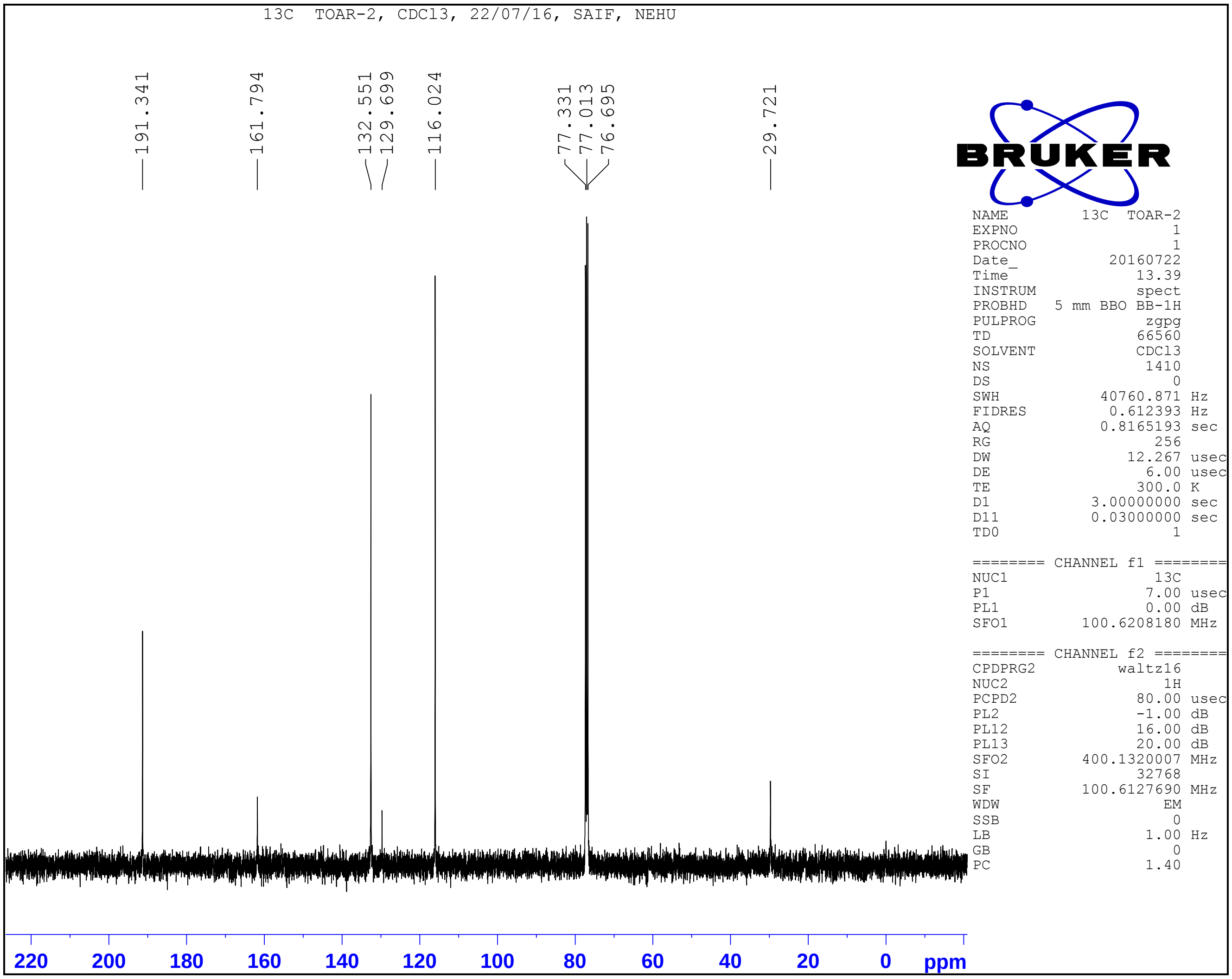

^{13}C NMR spectrum of compound **3'b**

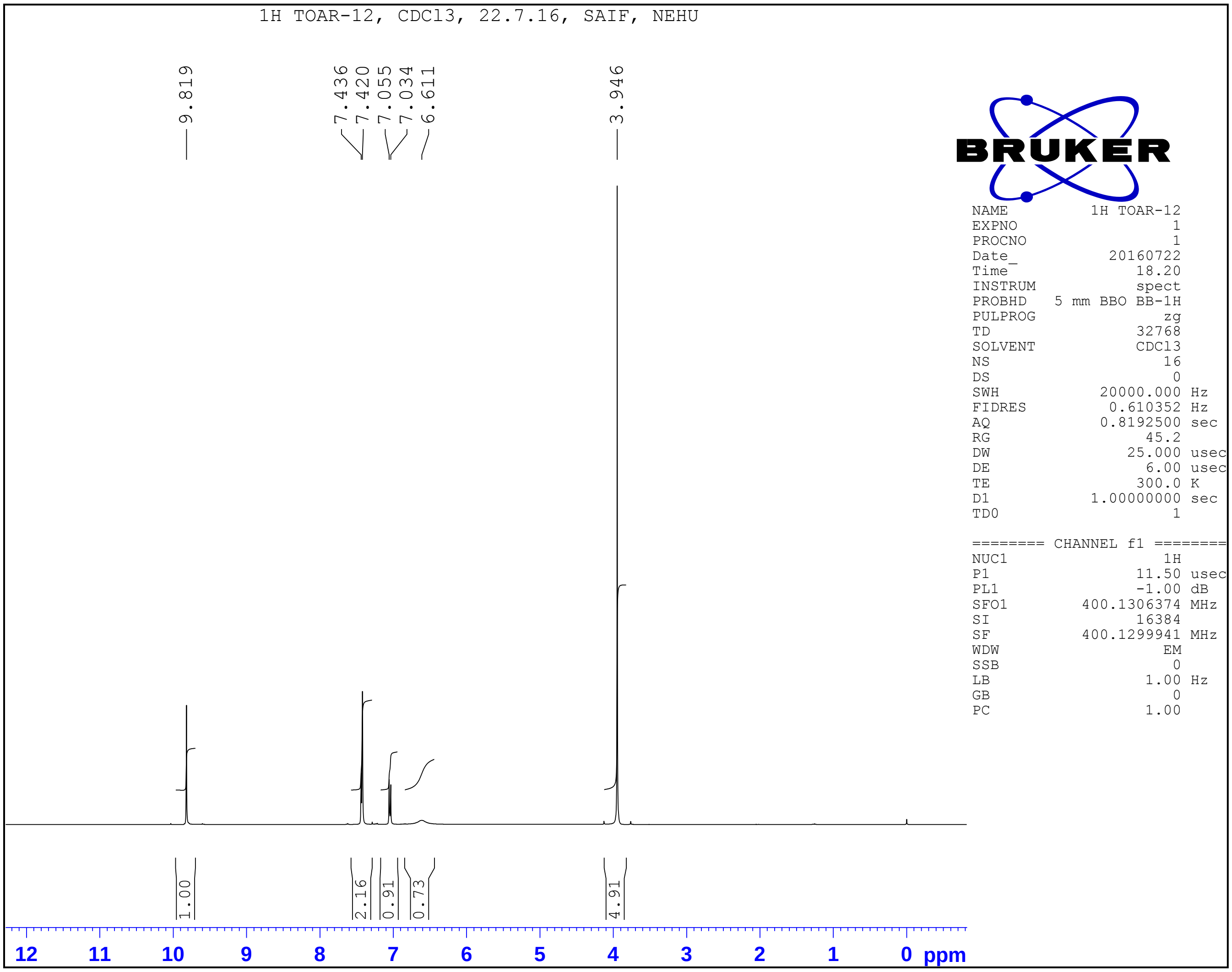

^{1}H NMR spectrum of compound **3'l**

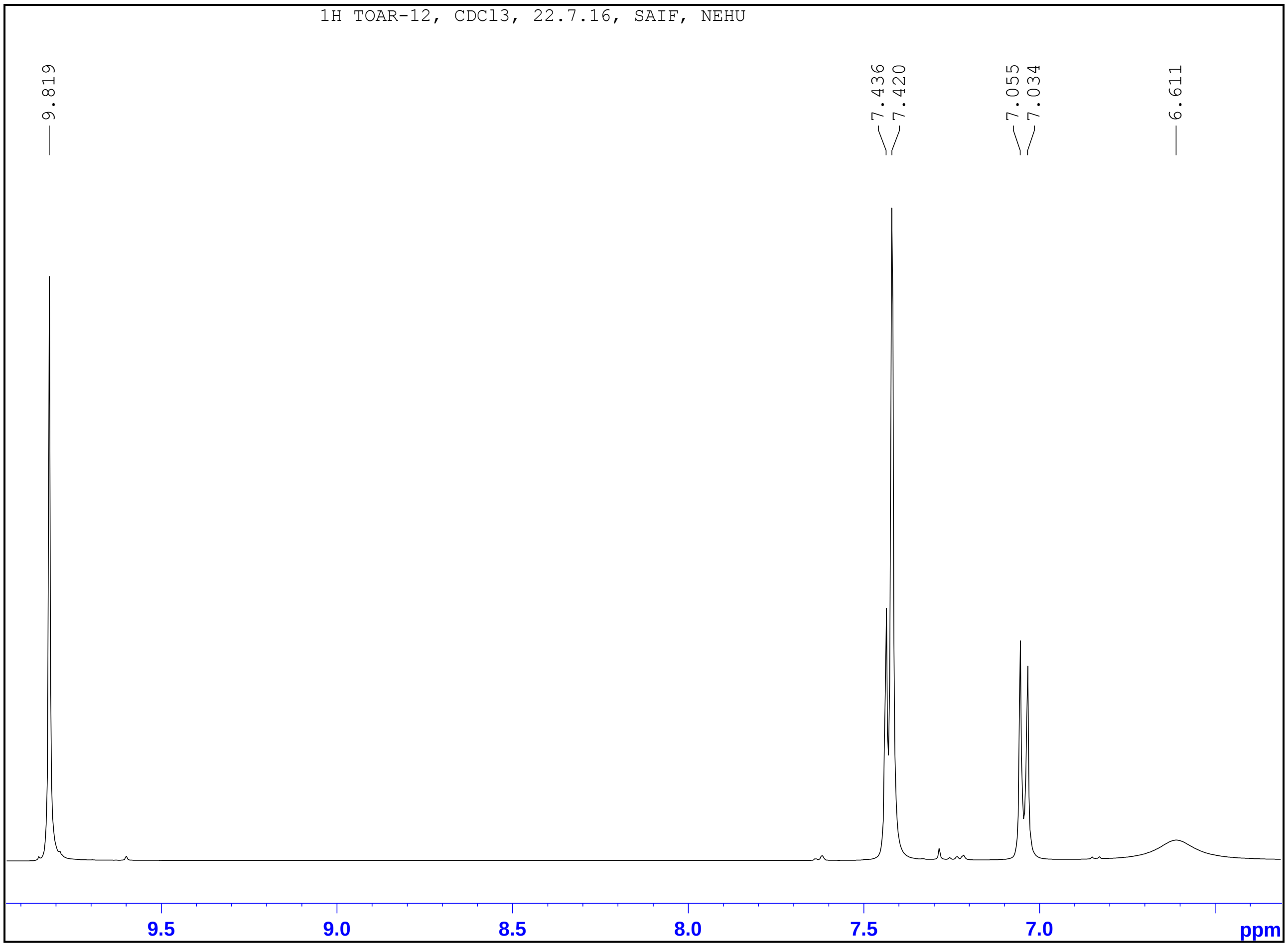

^{1}H NMR spectrum of compound **3'l**

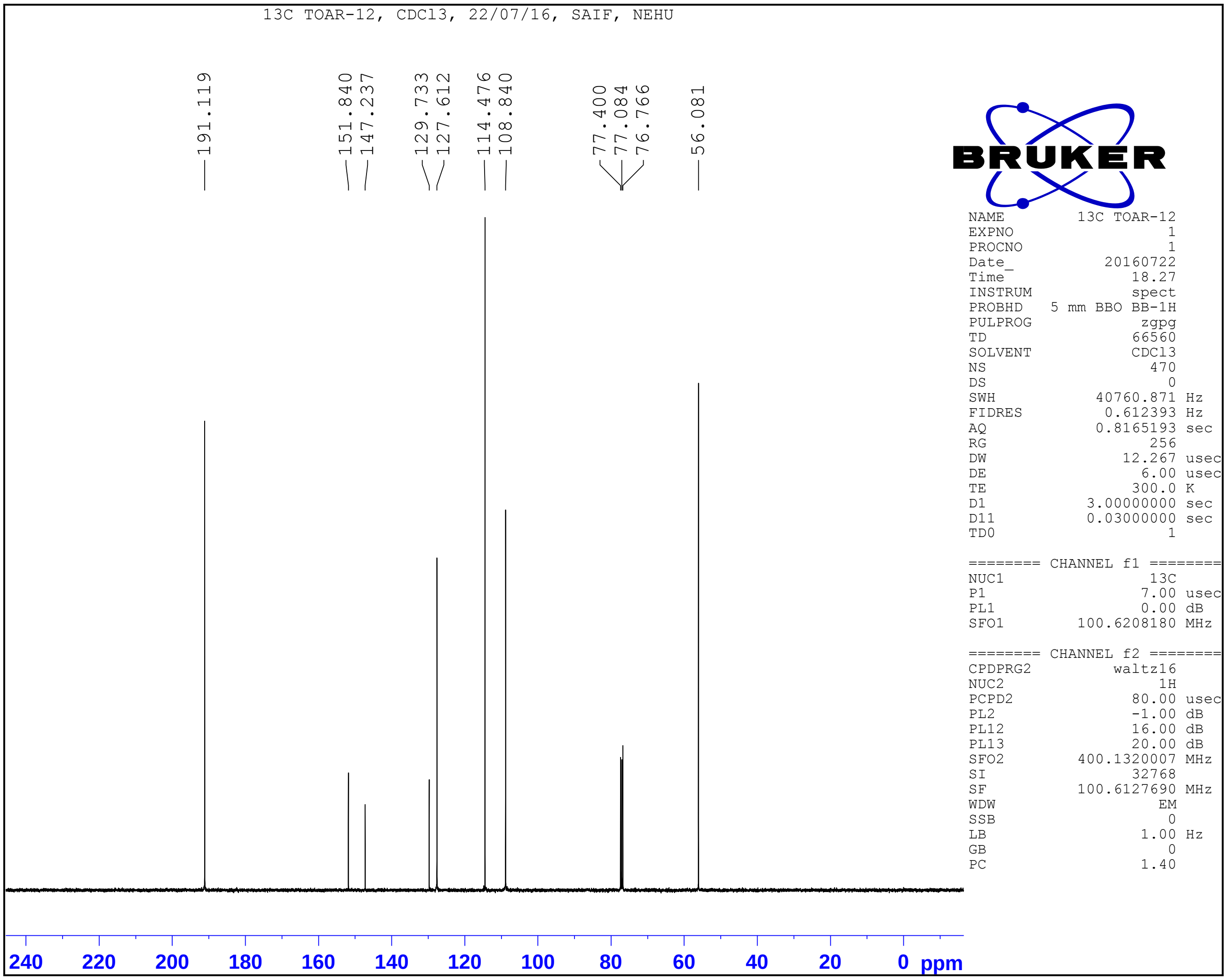

^{1}H NMR spectrum of compound **3'l**

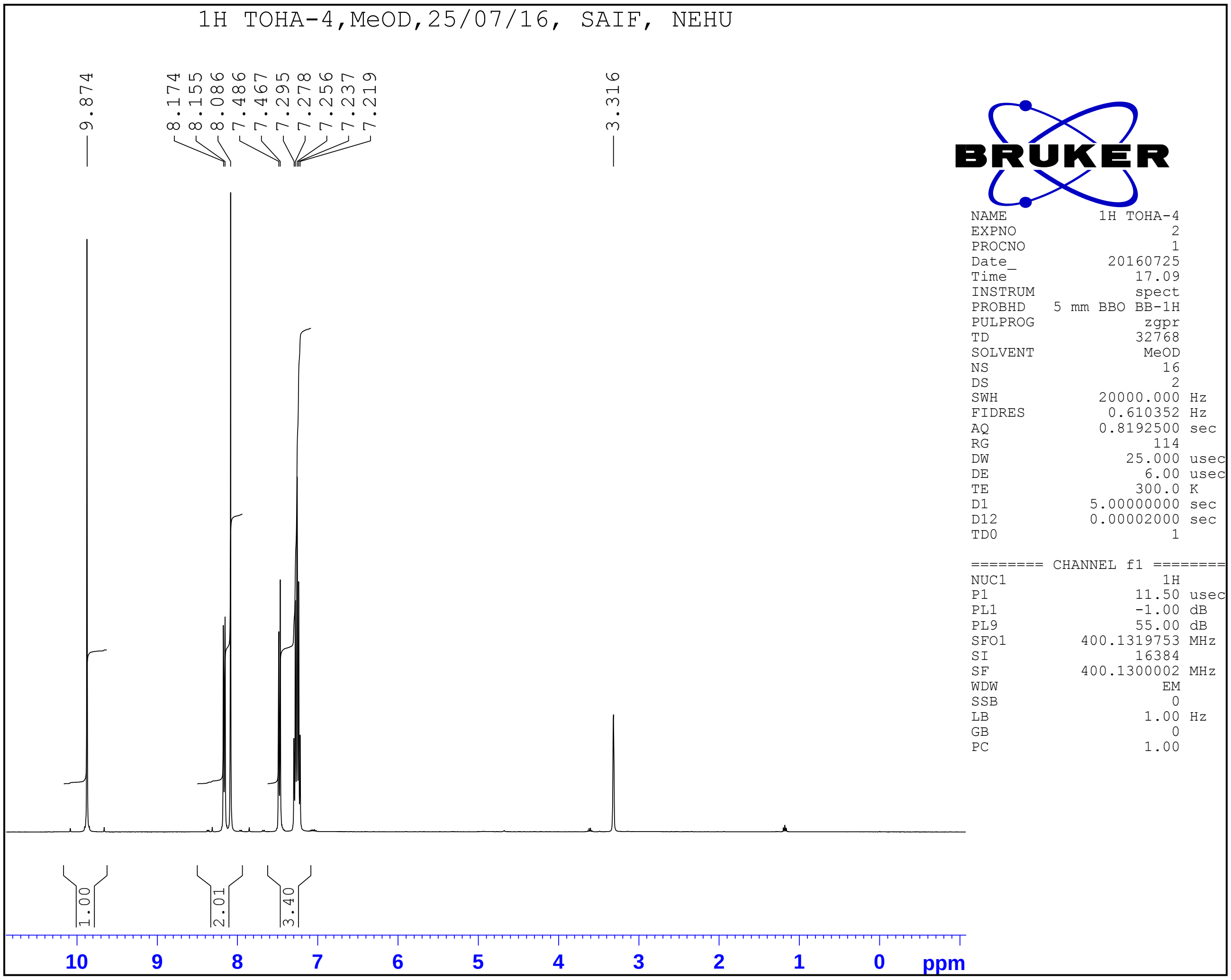

^{1}H NMR spectrum of compound **3''d**

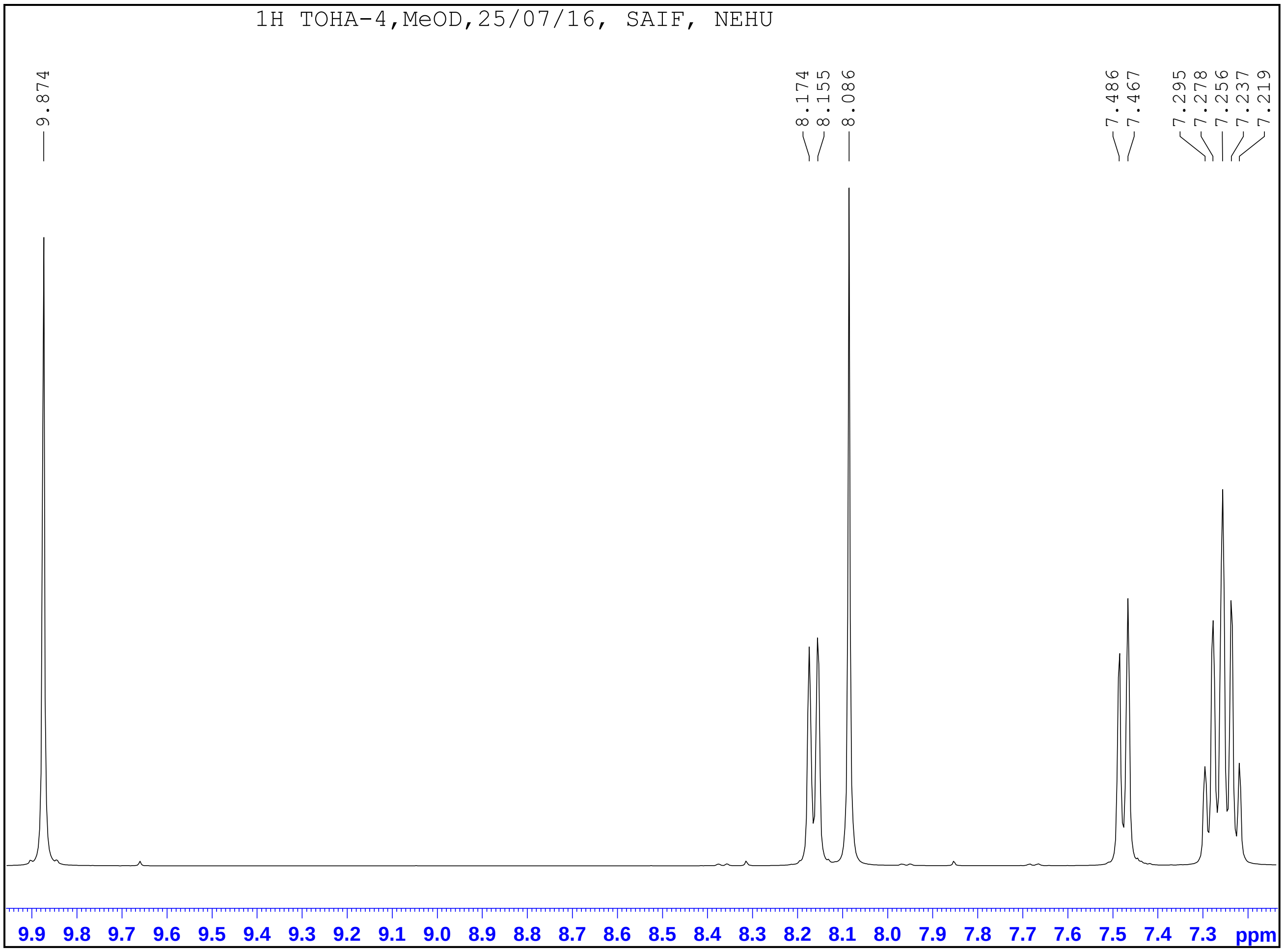

^{1}H NMR spectrum of compound **3''d**

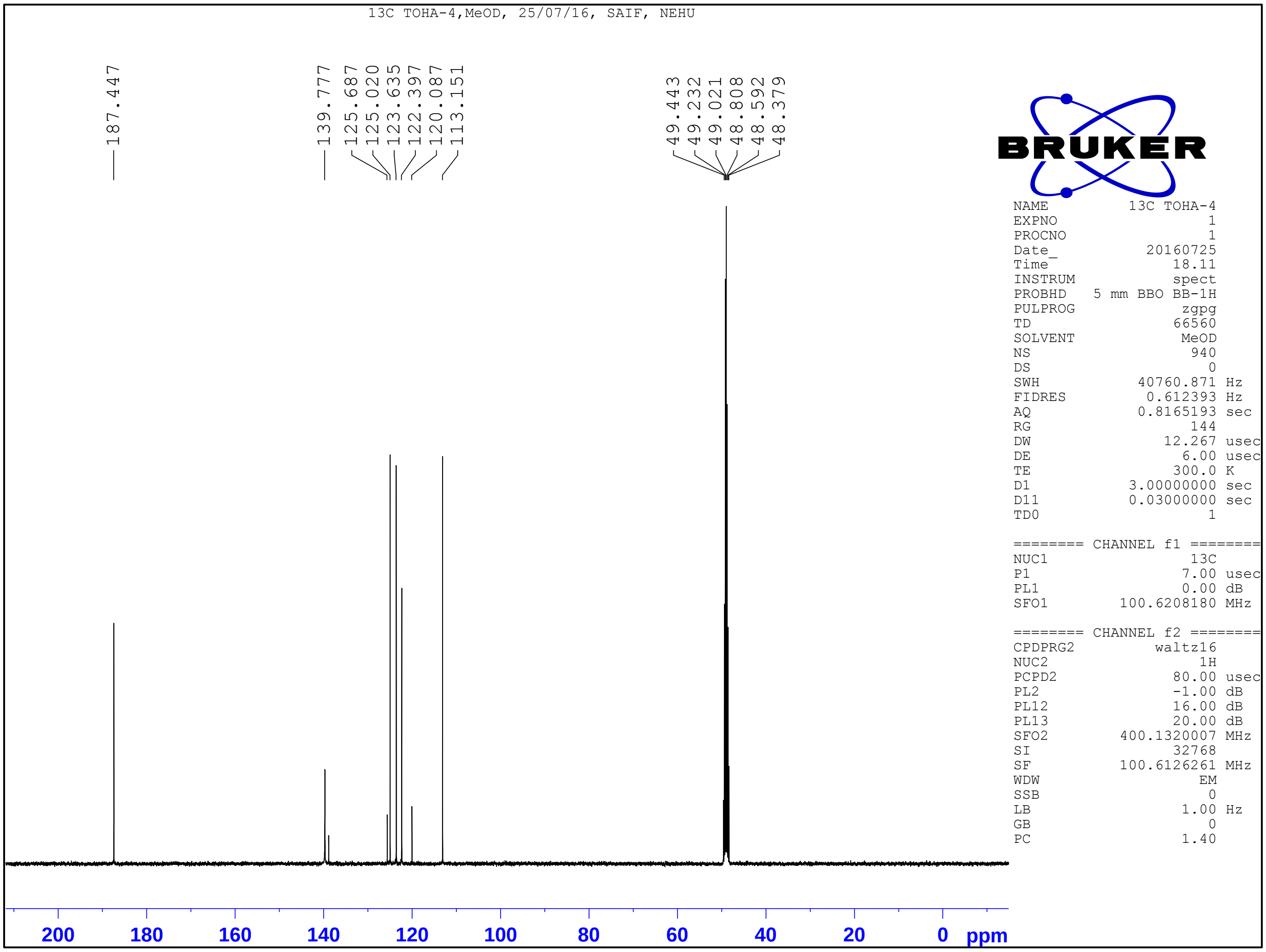

^{13}C NMR spectrum of compound **3''d**
